# Supplementary material for: A dual diffusion model enables 3D molecule generation and lead optimization based on target pockets
Source: Nat Commun. 2024 Mar 26;15:2657. doi: 10.1038/s41467-024-46569-1 (PMC10965937; doi:10.1038/s41467-024-46569-1)
Supplement: Supplementary file 1 — Supplementary information [file 41467_2024_46569_MOESM1_ESM.pdf]

SUPPLEMENTARY DOCUMENT

A dual diffusion model enables 3D molecule generation and lead optimization based on target pockets

March 11, 2024

A Notations used in this paper

We provide notations used in this paper for easier reading.

Notations	Descriptions
t	Time step
x	The atom representation
r	The position of the atoms
A	adjacent matrix
$\mathbf{G}_0$	The ground truth molecule geometry
$\mathbf{G}_{1\ldots T}$	The latent information via diffusion
β_t	A fixed variance schedule
α_t	$\alpha_t = 1 - \beta_t$
$\bar{\alpha}_t$	$\bar{\alpha}_t = \prod_{s=1}^{t} 1 - \beta_s$
$\mathbf{d}_{ij}$	The Euclidean distance between atom i and atom j
e_{ij}	Edge between atom i and atom j
σ_t^2	User defined variance
σ_v	The standard deviation outputted by VAE encoder
μ_v	The mean outputted by VAE encoder
$\boldsymbol{\epsilon}_\theta$	Parameterized noise
$\boldsymbol{\epsilon}$	$\boldsymbol{\epsilon} = [\boldsymbol{\epsilon}^a, \boldsymbol{\epsilon}^r]$ where $\boldsymbol{\epsilon}^a \sim \mathcal{N}(0, I)$,$\boldsymbol{\epsilon}^r \sim \mathcal{N}(0, I)$
$\boldsymbol{s}_\theta$	Parameterized stein score
$\boldsymbol{\mu}_\theta$<br>z	Parameterized mean<br>Sample from $\mathcal{N}(0, I)$
ξ	Sample from $\mathcal{N}(0, I)$
ϕ	Neural networks
$q()$	Distribution of diffusion process
$p()$	Distribution of reverse process

Supplementary Table 1. Notations used in this paper.

B Proof of the diffusion model

We provide proofs for the derivation of several properties in the diffusion model. For a detailed explanation and discussion, we refer readers to [1].

B.1 Marginal distribution of the diffusion process

In the diffusion process, we have the marginal distribution of the data at any arbitrary time step t in a closed form:

$$q\left(\mathbf{G}_t \mid \mathbf{G}_0\right) = \mathcal{N}\left(\mathbf{G}_t; \sqrt{\bar{\alpha}_t}\mathbf{G}_0, \left(1 - \bar{\alpha}_t\right) I\right). \tag{1}$$

Recall the posterior $q\left(\mathbf{G}_t \mid \mathbf{G}_{t-1}\right)$ in Eq.1 (main document), we can obtain $\mathbf{G}_t$ using the reparameterization trick. A property of the Gaussian distribution is that if we add $\mathcal{N}(\mathbf{0}, \sigma_1^2\mathbf{I})$ and $\mathcal{N}(\mathbf{0}, \sigma_2^2\mathbf{I})$, the new distribution

is $\mathcal{N}(\mathbf{0},(\sigma_1^2+\sigma_2^2)\mathbf{I})$

$$
\begin{aligned}
\mathbf{G}_t &= \sqrt{\alpha_t}\mathbf{G}_{t-1}+\sqrt{1-\alpha_t}\epsilon_{t-1} \\
&= \sqrt{\alpha_t\alpha_{t-1}}\mathbf{G}_{t-2}+\sqrt{\alpha_t(1-\alpha_{t-1})}\epsilon_{t-2}+\sqrt{1-\alpha_t}\epsilon_{t-1} \\
&= \sqrt{\alpha_t\alpha_{t-1}}\mathbf{G}_{t-2}+\sqrt{1-\alpha_t\alpha_{t-1}}\bar{\epsilon}_{t-2} \\
&= \ldots \\
&= \sqrt{\bar{\alpha}_t}\mathbf{G}_0+\sqrt{1-\bar{\alpha}_t}\bar{\epsilon},
\end{aligned}
\tag{2}
$$

where $\alpha_t=1-\beta_t$, ϵ and $\hat{\epsilon}$ are sampled from independent standard Gaussian distributions.

B.2 The parameterized mean μ_θ

A learned Gaussian transitions $p_\theta\left(\mathbf{G}_{t-1}\mid\mathbf{G}_t\right)$ is devised to approximate the $q\left(\mathbf{G}_{t-1}\mid\mathbf{G}_t\right)$ of every time step: $p_\theta\left(\mathbf{G}_{t-1}\mid\mathbf{G}_t\right)=\mathcal{N}\left(\mathbf{G}_{t-1};\boldsymbol{\mu}_\theta\left(\mathbf{G}_t,t\right),\sigma_t^2 I\right)$. $\boldsymbol{\mu}_\theta$ is parameterized as follows:

$$
\boldsymbol{\mu}_\theta\left(\mathbf{G}_t,t\right)=\frac{1}{\sqrt{\alpha_t}}\left(\mathbf{G}_t-\frac{\beta_t}{\sqrt{1-\bar{\alpha}_t}}\boldsymbol{\epsilon}_\theta\left(\mathbf{G}_t,t\right)\right). \tag{3}
$$

The distribution $q\left(\mathbf{G}_{t-1}\mid\mathbf{G}_t\right)$ can be expanded by Bayes' rule:

$$
\begin{aligned}
q\left(\mathbf{G}_{t-1}\mid\mathbf{G}_t\right) &= q\left(\mathbf{G}_{t-1}\mid\mathbf{G}_t,\mathbf{G}_0,\right) \\
&= q\left(\mathbf{G}_t\mid\mathbf{G}_{t-1},\mathbf{G}_0\right)\frac{q\left(\mathbf{G}_{t-1}\mid\mathbf{G}_0\right)}{q\left(\mathbf{G}_t\mid\mathbf{G}_0\right)} \\
&= q\left(\mathbf{G}_t\mid\mathbf{G}_{t-1}\right)\frac{q\left(\mathbf{G}_{t-1}\mathbf{G}_0\right)}{q\left(\mathbf{G}_t\mid\mathbf{G}_0\right)} \\
&\propto \exp\left(-\frac{1}{2}\left(\frac{\left(\mathbf{G}_t-\sqrt{\alpha_t}\mathbf{G}_{t-1}\right)^2}{\beta_t}+\frac{\left(\mathbf{G}_{t-1}-\sqrt{\alpha_{t-1}}\mathbf{G}_0\right)^2}{1-\bar{\alpha}_{t-1}}-\frac{\left(\mathbf{G}_t-\sqrt{\alpha_t}\mathbf{G}_0\right)^2}{1-\bar{\alpha}_t}\right)\right) \\
&= \exp\left(-\frac{1}{2}\left(\left(\frac{\alpha_t}{\beta_t}+\frac{1}{1-\bar{\alpha}_{t-1}}\right)\mathbf{G}_{t-1}^2-\left(\frac{2\sqrt{\alpha_t}}{\beta_t}\mathbf{G}_t+\frac{2\sqrt{\alpha_{t-1}}}{1-\bar{\alpha}_{t-1}}\mathbf{G}_0\right)\mathbf{G}_{t-1}+C\left(\mathbf{G}_t,\mathbf{G}_0\right)\right)\right) \\
&\propto \exp(-\mathbf{G}_{t-1}^2+(\frac{\sqrt{\alpha_t}\left(1-\bar{\alpha}_{t-1}\right)}{1-\bar{\alpha}_t}\mathbf{G}_t+\frac{\sqrt{\bar{\alpha}_{t-1}}\beta_t}{1-\bar{\alpha}_t}\mathbf{G}_0)\mathbf{G}_{t-1}),
\end{aligned}
\tag{4}
$$

where $C\left(\mathbf{G}_t,\mathbf{G}_0\right)$ is a constant. We can find that $q\left(\mathbf{G}_{t-1}\mid\mathbf{G}_t\right)$ is also a Gaussian distribution. We assume that:

$$
q\left(\mathbf{G}_{t-1}\mid\mathbf{G}_t,\mathbf{G}_0\right)=\mathcal{N}\left(\mathbf{G}_{t-1};\tilde{\boldsymbol{\mu}}\left(\mathbf{G}_t,\mathbf{G}_0\right),\tilde{\beta}_t I\right), \tag{5}
$$

where $\tilde{\beta}_t = 1/\left(\frac{\alpha_t}{\beta_t} + \frac{1}{1-\bar{\alpha}_{t-1}}\right) = \frac{1-\bar{\alpha}_{t-1}}{1-\bar{\alpha}_t} \cdot \beta_t$ and $\tilde{\boldsymbol{\mu}}_t\left(\mathbf{G}_t, \mathbf{G}_0\right) = \left(\frac{\sqrt{\alpha_t}}{\beta_t}\mathbf{G}_t + \frac{\sqrt{\bar{\alpha}_{t-1}}}{1-\bar{\alpha}_{t-1}}\mathbf{G}_0\right) / \left(\frac{\alpha_t}{\beta_t} + \frac{1}{1-\bar{\alpha}_{t-1}}\right) = \frac{\sqrt{\alpha_t}(1-\bar{\alpha}_{t-1})}{1-\bar{\alpha}_t}\mathbf{G}_t + \frac{\sqrt{\bar{\alpha}_{t-1}}\beta_t}{1-\bar{\alpha}_t}\mathbf{G}_0$.

From Eq. 2, we have $\mathbf{G}_t == \sqrt{\bar{\alpha}_t}\mathbf{G}_0 + \sqrt{1-\bar{\alpha}_t}\bar{\epsilon}$. We take this into $\tilde{\boldsymbol{\mu}}$:

$$\begin{aligned}
\tilde{\boldsymbol{\mu}}_t &= \frac{\sqrt{\alpha_t}\left(1-\bar{\alpha}_{t-1}\right)}{1-\bar{\alpha}_t}\mathbf{x}_t + \frac{\sqrt{\bar{\alpha}_{t-1}}\beta_t}{1-\bar{\alpha}_t}\frac{1}{\sqrt{\bar{\alpha}_t}}\left(\mathbf{x}_t - \sqrt{1-\bar{\alpha}_t}\epsilon_t\right) \\
&= \frac{1}{\sqrt{\alpha_t}}\left(\mathbf{x}_t - \frac{\beta_t}{\sqrt{1-\bar{\alpha}_t}}\epsilon_t\right)
\end{aligned} \tag{6}$$

$\boldsymbol{\mu}_\theta$ is designed to model $\tilde{\boldsymbol{\mu}}$. Therefore, $\boldsymbol{\mu}_\theta$ has the same formulation as $\tilde{\boldsymbol{\mu}}$ but parameterizes ϵ:

$$\boldsymbol{\mu}_\theta\left(\mathbf{G}_t, t\right) = \frac{1}{\sqrt{\alpha_t}}\left(\mathbf{G}_t - \frac{\beta_t}{\sqrt{1-\bar{\alpha}_t}}\boldsymbol{\epsilon}_\theta\left(\mathbf{G}_t, t\right)\right). \tag{7}$$

B.3 Decompose atomic coordinates to pairwise distances

In order to achieve the equivariance of the atomic coordinates in 3D space, we attempt to decompose them to pairwise distances.

$$\begin{aligned}
&g(\mathbf{r}) = \mathbf{d}, \\
&\log p_\theta(\mathbf{r}) \triangleq f \circ g(\mathbf{r}) = f(\mathbf{d})
\end{aligned} \tag{8}$$

Where $g : \mathbb{R}^{n\times 3} \to \mathbb{R}^{|E|\times 1}$ denotes a function that maps the n atomic coordinates to $|E|$ interatomic distances and $\mathbb{R}^{|E|} \to \mathbb{R}$ is a neural network that estimates the log density of a molecule based on the interatomic distances $\mathbf{d}$.

$$\begin{aligned}
\forall i, \mathbf{s}_\theta(\mathbf{r}_i) = \frac{\partial f(\mathbf{d})}{\partial \mathbf{r}_i} &= \sum_{(i,j), e_{ij}\in E} \frac{\partial f(\mathbf{d})}{\partial d_{ij}} \cdot \frac{\partial d_{ij}}{\partial \mathbf{r}_i} \\
&= \sum_{j\in N(i)} \frac{1}{d_{ij}} \cdot \frac{\partial f(\mathbf{d})}{\partial d_{ij}} \cdot (\mathbf{r}_i - \mathbf{r}_j) \\
&= \sum_{j\in N(i)} \frac{1}{d_{ij}} \cdot \mathbf{s}_\theta(\mathbf{d}_{ij}) \cdot (\mathbf{r}_i - \mathbf{r}_j)
\end{aligned} \tag{9}$$

We refer readers to [3] for details.

B.4 The ELBO objective

It is hard to directly calculate conditional log likelihood of the data. Instead, we can derive its ELBO objective for optimizing. For simplicity, we denote the 3D ligand geometry $\mathbf{G}^L$ as $\mathbf{G}$

$$
\begin{aligned}
\mathbb{E}\left[-\log p_\theta\left(\mathbf{G}|\mathbf{G}^P\right)\right] &= -\mathbb{E}_{q(\mathbf{G}_0)} \log\left(\int p_\theta\left(\mathbf{G}_{0:T}|\mathbf{G}^P\right) d\mathbf{G}_{1:T}\right) \\
&= -\mathbb{E}_{q(\mathbf{G}_0)} \log\left(\int q\left(\mathbf{G}_{1:T} \mid \mathbf{G}_0\right) \frac{p_\theta\left(\mathbf{G}_{0:T}|\mathbf{G}^P\right)}{q\left(\mathbf{G}_{1:T} \mid \mathbf{G}_0\right)} d\mathbf{G}_{1:T}\right) \\
&= -\mathbb{E}_{q(\mathbf{G}_0)} \log\left(\mathbb{E}_{q(\mathbf{G}_{1:T}|\mathbf{G}_0)} \frac{p_\theta\left(\mathbf{G}_{0:T}|\mathbf{G}^P\right)}{q\left(\mathbf{G}_{1:T} \mid \mathbf{G}_0\right)}\right) \\
&\leq -\mathbb{E}_{q(\mathbf{G}_{0:T})} \log \frac{p_\theta\left(\mathbf{G}_{0:T}|\mathbf{G}^P\right)}{q\left(\mathbf{G}_{1:T} \mid \mathbf{G}_0\right)} \\
&= \mathbb{E}_{q(\mathbf{G}_{0:T})}\left[\log \frac{q\left(\mathbf{G}_{1:T} \mid \mathbf{G}_0\right)}{p_\theta\left(\mathbf{G}_{0:T}|\mathbf{G}^P\right)}\right]
\end{aligned} \tag{10}
$$

Then we further derive the conditional ELBO objective:

$$
\begin{aligned}
&\mathbb{E}_{q(\mathbf{G}_{0:T})}\left[\log \frac{q\left(\mathbf{G}_{1:T} \mid \mathbf{G}_0\right)}{p_\theta\left(\mathbf{G}_{0:T} | \mathbf{G}^P\right)}\right] \\
=&\mathbb{E}_q\left[\log \frac{\prod_{t=1}^T q\left(\mathbf{G}_t \mid \mathbf{G}_{t-1}\right)}{p_\theta\left(\mathbf{G}_T | \mathbf{G}^P\right) \prod_{t=1}^T p_\theta\left(\mathbf{G}_{t-1} \mid \mathbf{G}_t, \mathbf{G}^P\right)}\right] \\
=&\mathbb{E}_q\left[-\log p_\theta\left(\mathbf{G}_T | \mathbf{G}^P\right)+\sum_{t=1}^T \log \frac{q\left(\mathbf{G}_t \mid \mathbf{G}_{t-1}\right)}{p_\theta\left(\mathbf{G}_{t-1} \mid \mathbf{G}_t, \mathbf{G}^P\right)}\right] \\
=&\mathbb{E}_q\left[-\log p_\theta\left(\mathbf{G}_T | \mathbf{G}^P\right)+\sum_{t=2}^T \log \frac{q\left(\mathbf{G}_t \mid \mathbf{G}_{t-1}\right)}{p_\theta\left(\mathbf{G}_{t-1} \mid \mathbf{G}_t, \mathbf{G}^P\right)}+\log \frac{q\left(\mathbf{G}_1 \mid \mathbf{G}_0\right)}{p_\theta\left(\mathbf{G}_0 \mid \mathbf{G}_1, \mathbf{G}^P\right)}\right] \\
=&\mathbb{E}_q\left[-\log p_\theta\left(\mathbf{G}_T | \mathbf{G}^P\right)+\sum_{t=2}^T \log \left(\frac{q\left(\mathbf{G}_{t-1} \mid \mathbf{G}_t, \mathbf{G}_0\right)}{p_\theta\left(\mathbf{G}_{t-1} \mid \mathbf{G}_t, \mathbf{G}^P\right)} \cdot \frac{q\left(\mathbf{G}_t \mid \mathbf{G}_0\right)}{q\left(\mathbf{G}_{t-1} \mid \mathbf{G}_0\right)}\right)+\log \frac{q\left(\mathbf{G}_1 \mid \mathbf{G}_0\right)}{p_\theta\left(\mathbf{G}_0 \mid \mathbf{G}_1, \mathbf{G}^P\right)}\right] \\
=&\mathbb{E}_q\left[-\log p_\theta\left(\mathbf{G}_T | \mathbf{G}^P\right)+\sum_{t=2}^T \log \frac{q\left(\mathbf{G}_{t-1} \mid \mathbf{G}_t, \mathbf{G}_0\right)}{p_\theta\left(\mathbf{G}_{t-1} \mid \mathbf{G}_t, \mathbf{G}^P\right)}+\sum_{t=2}^T \log \frac{q\left(\mathbf{G}_t \mid \mathbf{G}_0\right)}{q\left(\mathbf{G}_{t-1} \mid \mathbf{G}_0\right)}+\log \frac{q\left(\mathbf{G}_1 \mid \mathbf{G}_0\right)}{p_\theta\left(\mathbf{G}_0 \mid \mathbf{G}_1, \mathbf{G}^P\right)}\right] \\
=&\mathbb{E}_q\left[-\log p_\theta\left(\mathbf{G}_T | \mathbf{G}^P\right)+\sum_{t=2}^T \log \frac{q\left(\mathbf{G}_{t-1} \mid \mathbf{G}_t, \mathbf{G}_0\right)}{p_\theta\left(\mathbf{G}_{t-1} \mid \mathbf{G}_t, \mathbf{G}^P\right)}+\log \frac{q\left(\mathbf{G}_T \mid \mathbf{G}_0\right)}{q\left(\mathbf{G}_1 \mid \mathbf{G}_0\right)}+\log \frac{q\left(\mathbf{G}_1 \mid \mathbf{G}_0\right)}{p_\theta\left(\mathbf{G}_0 \mid \mathbf{G}_1, \mathbf{G}^P\right)}\right] \\
=&\mathbb{E}_q\left[\log \frac{q\left(\mathbf{G}_T \mid \mathbf{G}_0\right)}{p_\theta\left(\mathbf{G}_T | \mathbf{G}^P\right)}+\sum_{t=2}^T \log \frac{q\left(\mathbf{G}_{t-1} \mid \mathbf{G}_t, \mathbf{G}_0\right)}{p_\theta\left(\mathbf{G}_{t-1} \mid \mathbf{G}_t, \mathbf{G}^P\right)}-\log p_\theta\left(\mathbf{G}_0 \mid \mathbf{G}_1, \mathbf{G}^P\right)\right] \\
=&\mathbb{E}_q\Bigg[\underbrace{D_{\mathrm{KL}}\left(q\left(\mathbf{G}_T \mid \mathbf{G}_0\right) \| p_\theta\left(\mathbf{G}_T | \mathbf{G}^P\right)\right)}_{L_T}+ \\
&\sum_{t=2}^T \underbrace{D_{\mathrm{KL}}\left(q\left(\mathbf{G}_{t-1} \mid \mathbf{G}_t, \mathbf{G}_0\right) \| p_\theta\left(\mathbf{G}_{t-1} \mid \mathbf{G}_t, \mathbf{G}^P\right)\right)}_{L_t} \underbrace{-\log p_\theta\left(\mathbf{G}_0 \mid \mathbf{G}_1, \mathbf{G}^P\right)}_{L_0}\Bigg]
\end{aligned}
\tag{11}
$$

C Experiment details

C.1 Implementation

In this section, we introduce the implementation details of PMDM. Both the local and global ligand EGNNs of PMDM consist of 3 layers and the hidden feature dimension is 128. The protein SchNet of PMDM consists of 3 layers and the hidden feature dimension is 128. We set the local radius τ_l as 3Å which could include almost all the chemical bonds and the global radius τ_g as 6Å. We trained PMDM by Adam optimizer on 64 V100 cards for 500 epochs with a learning rate of 0.001. The batch size is 16. We took around 7 hours to train PMDM.

C.2 Ablation study

We remove the cross-attention layer, local equivariant kernel and global equivariant kernel to observe the performance degradation of PMDM. We observe that the Vina score will drop if we remove any module. Interestingly, QED and SA increase if we remove the cross-attantion (CA) layer, indicating that PMDM can concentrate on the molecule itself if it fuses less protein information. Compared with PMDM without local kernel, PMDM surpasses it on all matrics except for the sampling speed. All the deformed PMDM exceed the test set. We do not report the results of PMDM without global kernel since we find that PMDM cannot generate valid molecules if we remove this indispensable module.

Supplementary Table 2. Albation studies.

Methods	Vina Score (kcal/mol) ↓	High Affinity ↑	QED ↑	SA ↑	Lipinski ↑	LogP	Diversity	Time (seconds) ↓
PMDM	**-7.572** ± 2.50	**0.628**	0.594 ± 0.12	0.611 ± 0.16	**4.975** ± 0.16	0.301 ± 1.01	0.709 ± 0.10	906 ± 110
PMDM w.o. CA	-7.515 ± 2.26	0.620	**0.606** ± 0.14	**0.689** ± 0.14	4.951 ± 0.24	0.514 ± 0.97	0.704 ± 0.09	636 ± 680
PMDM w.o. local	-7.496 ± 2.37	0.598	0.582 ± 0.14	0.598 ± 0.13	4.917 ± 0.33	0.862 ± 1.46	0.720 ± 0.08	**585** ± 529
Test set	-7.024	-	0.466	0.725	1.413	0.929	-	-

Since PMDM without CA performs better than PMDM on QED and SA, we further investigate whether PMDM without CA can generate molecules binding to the SARS-CoV-2 main protease (M^{pro}) with high SA scores and rational medicinal property values. We also generate 40000 molecules and calculate their Vina, SA, QED, logP and Lipinski rules to compare with PMDM. As demonstrated in Figure 1, we found that PMDM with CA outperformed PMDM without CA on Vina and SA, indicating that it can generate more potent and feasible inhibitors for M^{pro}. On the other hand, PMDM without CA achieved higher QED scores than PMDM with CA, suggesting that it can generate more drug-like molecules. However, both methods generated most molecules with reasonable logP values, and PMDM with CA generated more molecules that satisfied the Lipinski rules than PMDM without CA. Therefore, we conclude that PMDM with CA is a more effective method for generating molecules that can target M^{pro}.

D Algorithms of PMDM

We describe the training and sampling algorithms in this section. To ensure the invariance of ϵ, we introduce zero center of mass (COM) from the previous work[2] to achieve invariance for $p(\mathbf{G}_T)$. By extending the approximation of $p(\mathbf{G}_T)$ from a standard Gaussian to an isotropic Gaussian, the ϵ is invariant to rotations and translations around the zero COM. Algorithm 1 and algorithm 2 display the complete training procedure and sampling procedure. For the training process, we diffuse the real ligand data and use the ELBO

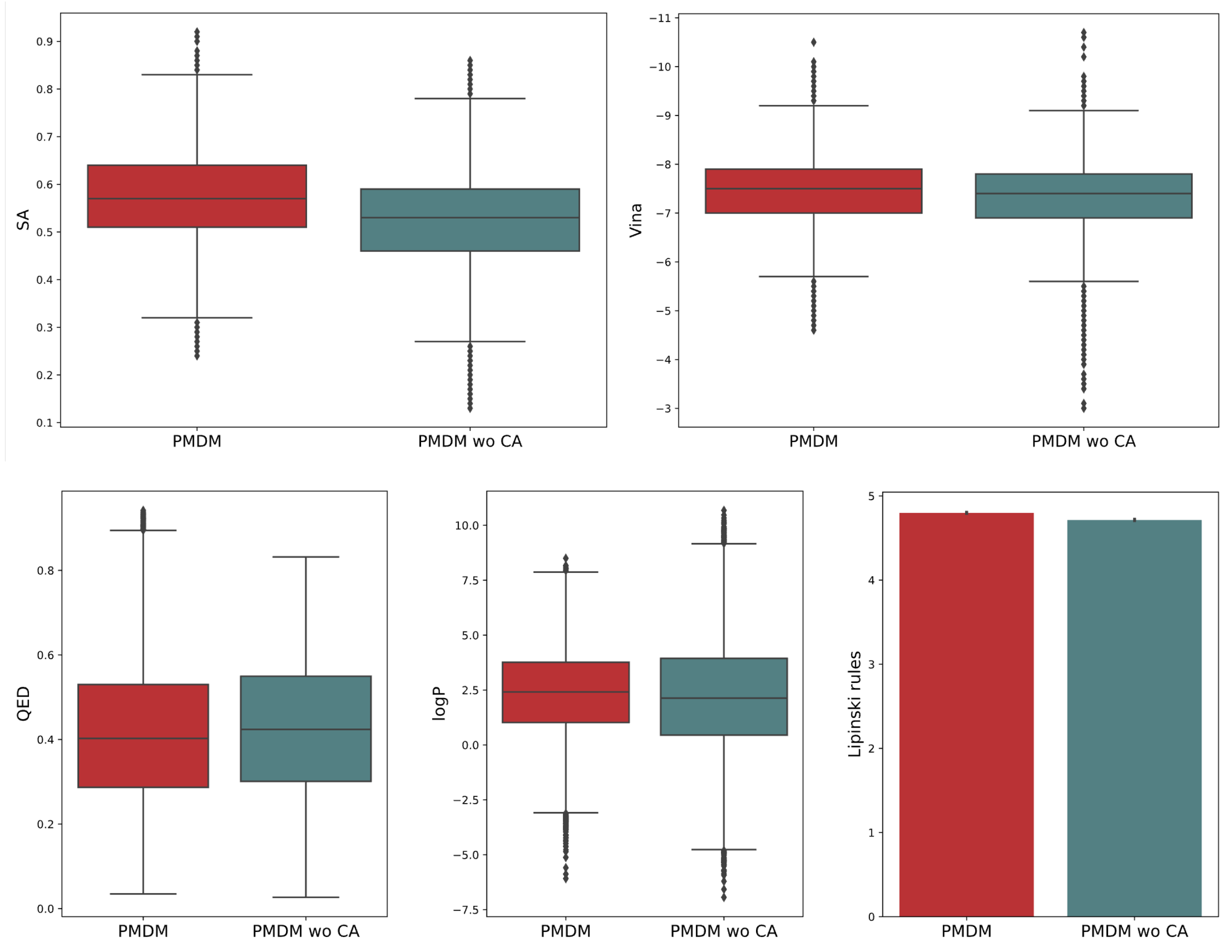

Supplementary Figure 1. Results comparison of PMDM and PMDM w.o. CA for generating 40000 molecules for SARS-CoV-2 main protease (M^{pro}) (n=10000 in each group; center line, median; box limits, upper and lower quartiles; upper line, maxima; whiskers, lower line, minima; 1.5 × interquartile range;). Source data are provided with this paper.

objective of the loss function to train the model. For the sampling process, the next less chaotic state $\mathbf{G}_{T-1}$ is generated by $\mathcal{N}(\mathbf{G}_T; \boldsymbol{\mu}_\theta, \sigma_T^2 I)$. The final molecule $\mathbf{G}_0$ is generated by progressively sample $\mathbf{G}_{t-1}$ for T times. Algorithm 3 demonstrates the sampling process given seed fragments which we employ this method for lead optimization. The seed fragment data will be diffused to be concatenated with the unknown (sampling) part, and we only update this part while keeping the seed fragment data fixed.

Algorithm 1 Training Process

Input: Ligand molecular geometry $\mathbf{G}^L(x^L, r^L)$, protein molecular geometry $\mathbf{G}^P(x^P, r^P)$, ligand encoder $\mathbf{SchNet}_l$, protein encoder $\mathbf{SchNet}_p$, co-attention later ϕ_{coa}, global equivariant neural networks ϕ_g, local neural networks ϕ_l

```
repeat
    x_0^L, x^P ~ q(x_0^L, x^P); r_0^L, r^P ~ q(r_0^L, r^P)
    t ~ U({1, ..., T}), ε^x ~ N(0, I), ε^r ~ N(0, I)
    G_t^L = sqrt(ᾱ_t) G_0^L + (1 − ᾱ_t) ε
    Move G_t^L to zero COM, move G^P to zero COM of G_t^L
    z_L = SchNet_L(G_t^L), h_P = SchNet_P(G^P)
    h_L = φ_coa(z_L, h_P)
    Prepare global edges e_g and local edges e_l
    s_θ(G_t^L, G^P, t) = φ_g(h_L, h_P, t, e_g) + φ_l(h_L, h_P, t, e_l)
    Take gradient descent step on
    ∇_θ ‖s_θ(G_t^L, G^P, t) − ∇_{G_t} log q_σ(G_t^L | G_0^L, G^P)‖^2
until Converged
```

Line-by-line in math notation:

repeat

$\mathbf{x}_0^L, \mathbf{x}^P \sim q\left(\mathbf{x}_0^L, \mathbf{x}^P\right); \mathbf{r}_0^L, \mathbf{r}^P \sim q\left(\mathbf{r}_0^L, \mathbf{r}^P\right)$

$t \sim \mathcal{U}(\{1, \ldots, T\})$, $\boldsymbol{\epsilon}^x \sim \mathcal{N}(0, I)$, $\boldsymbol{\epsilon}^r \sim \mathcal{N}(0, I)$

$\mathbf{G}_t^L = \sqrt{\bar{\alpha}_t}\mathbf{G}_0^L + (1 - \bar{\alpha}_t)\boldsymbol{\epsilon}$

Move $\mathbf{G}_t^L$ to zero COM, move $\mathbf{G}^P$ to zero COM of $\mathbf{G}_t^L$

$z_L = \mathrm{SchNet}_L(\mathbf{G}_t^L), h_P = \mathrm{SchNet}_P(\mathbf{G}^P)$

$h_L = \phi_{coa}(z_L, h_P)$

Prepare global edges e_g and local edges e_l

$\boldsymbol{s}_\theta\left(\mathbf{G}_t^L, \mathbf{G}^P, t\right) = \phi_g(h_L, h_P, t, e_g) + \phi_l(h_L, h_P, t, e_l)$

Take gradient descent step on
$\nabla_\theta \left\|\boldsymbol{s}_\theta\left(\mathbf{G}_t^L, \mathbf{G}^P, t\right) - \nabla_{\mathbf{G}_t} \log q_\sigma(\mathbf{G}_t^L \mid \mathbf{G}_0^L, \mathbf{G}^P)\right\|^2$

until Converged

Algorithm 2 Sampling Process

Input: Ligand encoder $\mathbf{SchNet}_l$, protein encoder $\mathbf{SchNet}_p$, co-attention later ϕ_{coa}, global equivariant neural networks ϕ_g, local neural networks ϕ_l

Output: the molecular coordinates r and atom types x

Sample $\mathbf{G}_t^L \sim \mathcal{N}(0, I)$

for $t = 1 ... T$ **do**

Sample $\xi \sim \mathcal{N}(0, I)$ if $t > 1$, else $\xi = \mathbf{0}$

Move $\mathbf{G}_t^L$ to zero COM, move $\mathbf{G}^P$ to zero COM of $\mathbf{G}_t^L$

$z_L = \mathrm{SchNet}_L(\mathbf{G}_t^L), h_P = \mathrm{SchNet}_P(\mathbf{G}^P)$

$h_L = \phi_{coa}(z_L, h_P)$

Prepare global edges e_g and local edges e_l

Sample $z_v \sim \mathcal{N}(0, I)$

$\boldsymbol{s}_\theta\left(\mathbf{G}_t^L, \mathbf{G}^P, t\right) = \phi_g(h_L, h_P, t, e_g) + \phi_l(h_L, h_P, t, e_l)$

$\boldsymbol{\mu}_\theta\left(\mathbf{G}_t^L, \mathbf{G}^P, t\right) = \frac{1}{\sqrt{1-\beta_t}}\left(\mathbf{G}_t + \beta_t \boldsymbol{s}_\theta\left(\mathbf{G}_t^L, \mathbf{G}^P, t\right)\right)$

$\mathbf{G}_{t-1}^L = \boldsymbol{\mu}_\theta\left(\mathbf{G}_t^L, \mathbf{G}^P, t\right) + \sigma_t \xi$

end for

return $\mathbf{G}_0^L$

Algorithm 3 Sampling Process given seed fragments

Input: The seed fragment $\mathbf{G}^s$, ligand encoder $\mathbf{SchNet}_l$, protein encoder $\mathbf{SchNet}_p$, co-attention later ϕ_{coa}, global equivariant neural networks ϕ_g, local neural networks ϕ_l

Output: the molecular coordinates R and atom types A

Sample $\mathbf{G}_t^L \sim \mathcal{N}(0, I)$

for $t = 1...T$ **do**

  Sample $\xi \sim \mathcal{N}(0, I)$ if $t > 1$, else $\xi = \mathbf{0}$

  $\mathbf{G}_t^s = \sqrt{\bar{\alpha}_t}\mathbf{G}^s + (1 - \bar{\alpha}_t)\boldsymbol{\epsilon}$

  Combine the unknown part and seed fragment: $\mathbf{G}_t^{L,\text{full}} = [\mathbf{G}_t^L, \mathbf{G}_t^s]$

  Move $\mathbf{G}_t^{L,\text{full}}$ to zero COM, move $\mathbf{G}^P$ to zero COM of $\mathbf{G}_t^{L,\text{full}}$

  $z_L = \text{SchNet}_L(\mathbf{G}_t^{L,\text{full}}), h_P = \text{SchNet}_P(\mathbf{G}^P)$

  $h_L = \phi_{coa}(z_L, h_P)$

  Prepare global edges e_g and local edges e_l

  Sample $z_v \sim \mathcal{N}(0, I)$

  $\boldsymbol{s}_\theta\left(\mathbf{G}_t^{L,\text{full}}, \mathbf{G}^P, t\right) = \phi_g(h_L, h_P, t, e_g) + \phi_l(h_L, h_P, t, e_l)$

  $\boldsymbol{\mu}_\theta\left(\mathbf{G}_t^{L,\text{full}}, \mathbf{G}^P, t\right) = \frac{1}{\sqrt{1-\beta_t}}\left(\mathbf{G}_t + \beta_t \boldsymbol{s}_\theta\left(\mathbf{G}_t^{L,\text{full}}, \mathbf{G}^P, t\right)\right)$

  $\mathbf{G}_t^{L,\text{full}} = \boldsymbol{\mu}_\theta\left(\mathbf{G}_t^{L,\text{full}}, \mathbf{G}^P, t\right) + \sigma_t \xi$

end for

return $\mathbf{G}_t^{L,\text{full}} = [\mathbf{G}_0^L, \mathbf{G}^s]$

E Additional figures

E.1 Angle distributions of baseline methods

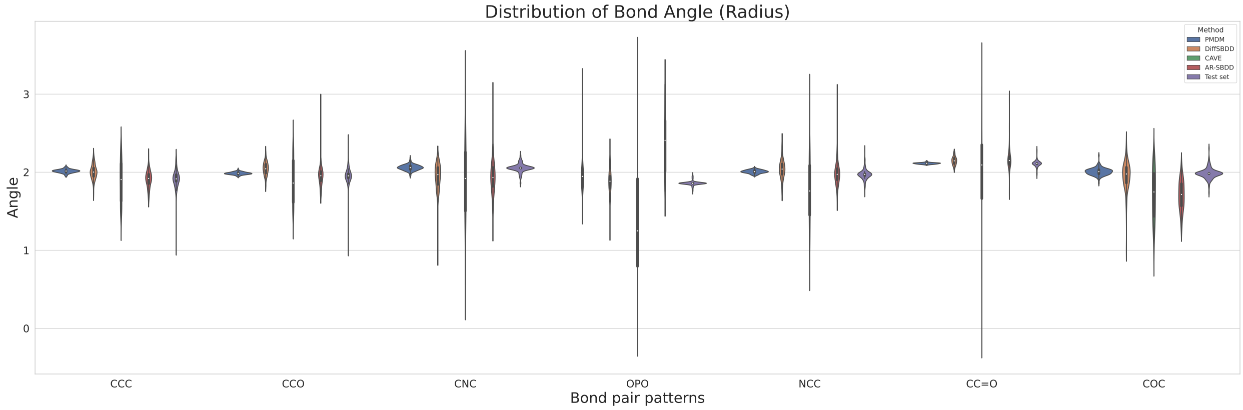

Supplementary Figure 2. Bond angle distributions comparison. Source data are provided with this paper.

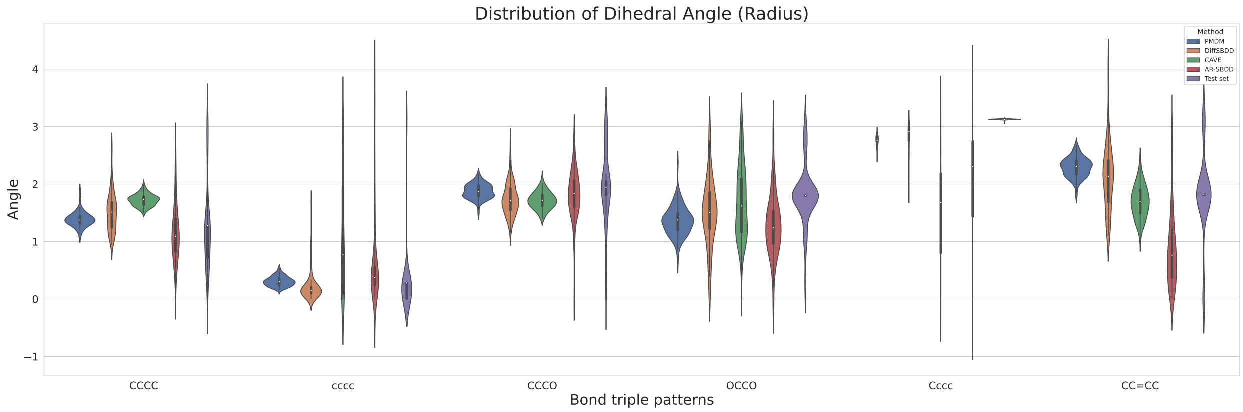

Supplementary Figure 3. Dihedral angle distributions comparison. Source data are provided with this paper.

E.2 Chemical space distributions of the baseline methods

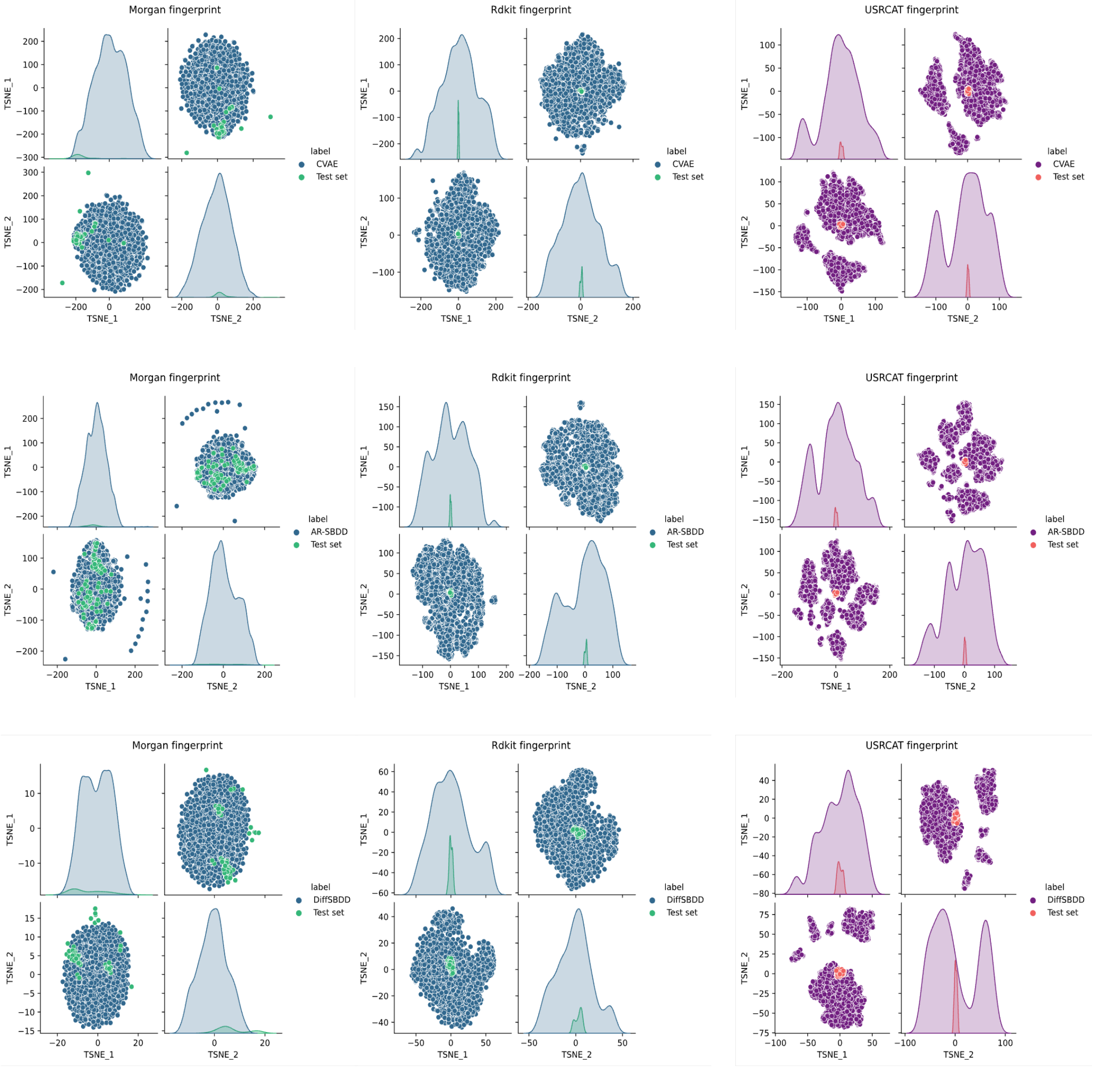

Supplementary Figure 4. The chemical space distribution visualization of baseline methods, including Morgan, RDKit, and USRCAT fingerprints using t-SNE in two-dimensional space. 3D chemical structure is measured by chemical descriptors.

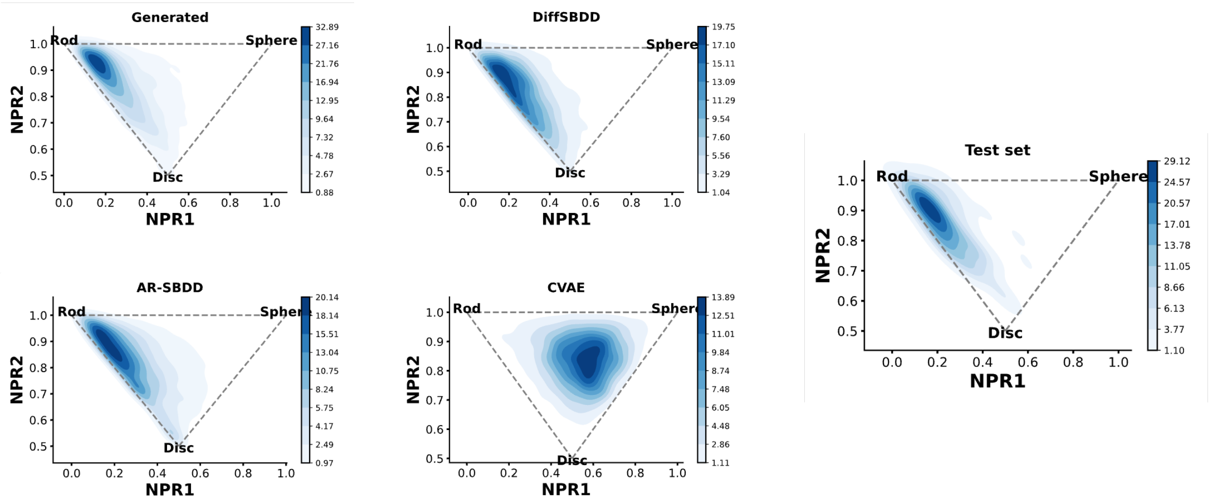

Supplementary Figure 5. Shape distribution of generated (left) and test set (right) molecules, which is visualized using the Normalized Principal Moment of Inertia ratios(NPR) descriptors.

E.3 Clash analysis

Since the pocket spatial information is treated as the condition, we keep the protein position fixed during the update of each layer of the equivariant kernel. The fixed protein positions can ensure the relative distance between the atoms of ligand and atoms of proteins, which could avoid the ligand clashes with the protein. We select three examples in Figure 7.

The generated molecules shown in the left part of Figure 7 are inclined to clash with the protein surface, although we initialize the ligand position in the center of the protein. When we treat the ligand and the protein as a whole pocket to input to the equivariant graph neural network and fix the protein positions, the model could learn the relative distances between ligands and proteins. The generated molecules do not prone to clash with the protein surfaces, which are shown in the right part of Figure 7.

E.4 Samples generated by PMDM

We provide more visualizations of generated samples which are trained on CrossDocked dataset in Figure 8 and Figure 9.

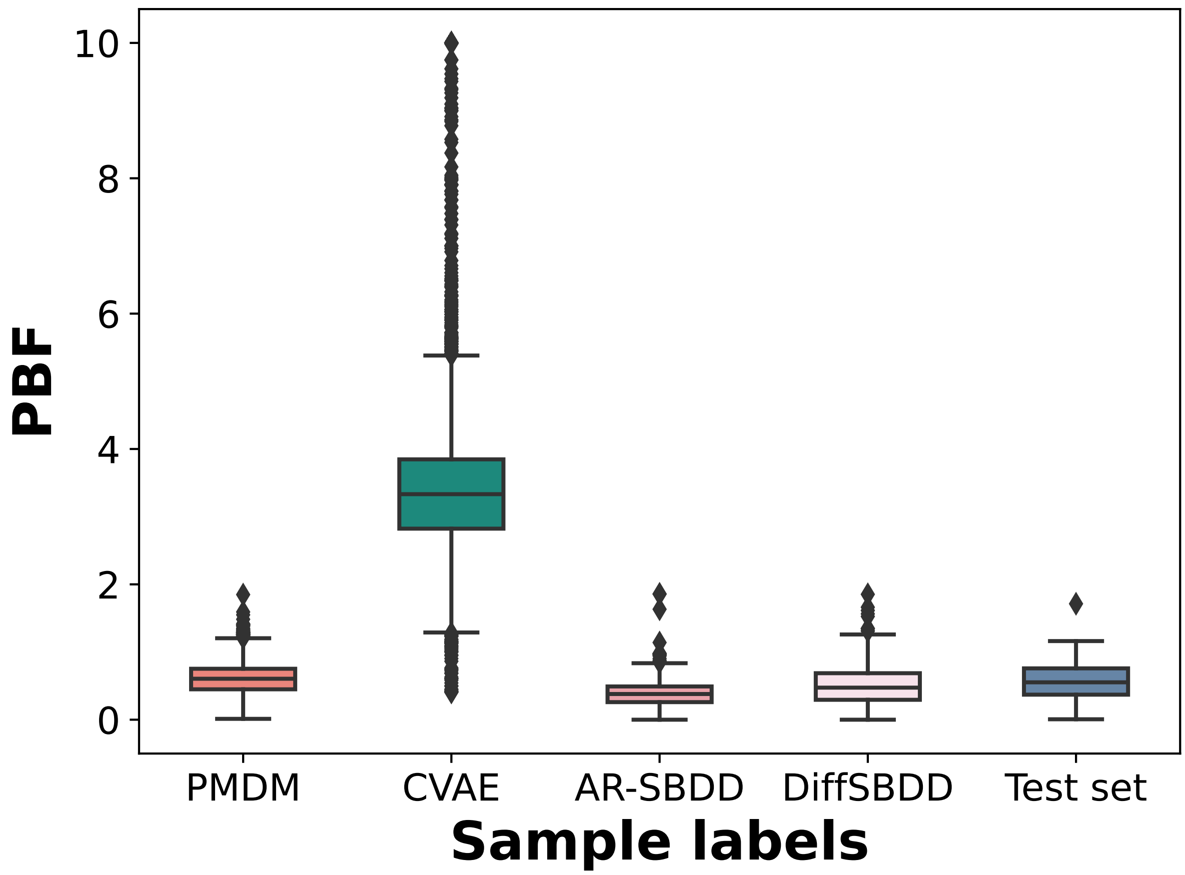

Supplementary Figure 6. The Plane of Best Fit (PBF) descriptor values of all the methods and test set (n=10000 for all the methods, n=100 for test set; center line, median; box limits, upper and lower quartiles; upper line, maxima; whiskers, lower line, minima; 1.5 × interquartile range;). Source data are provided with this paper.

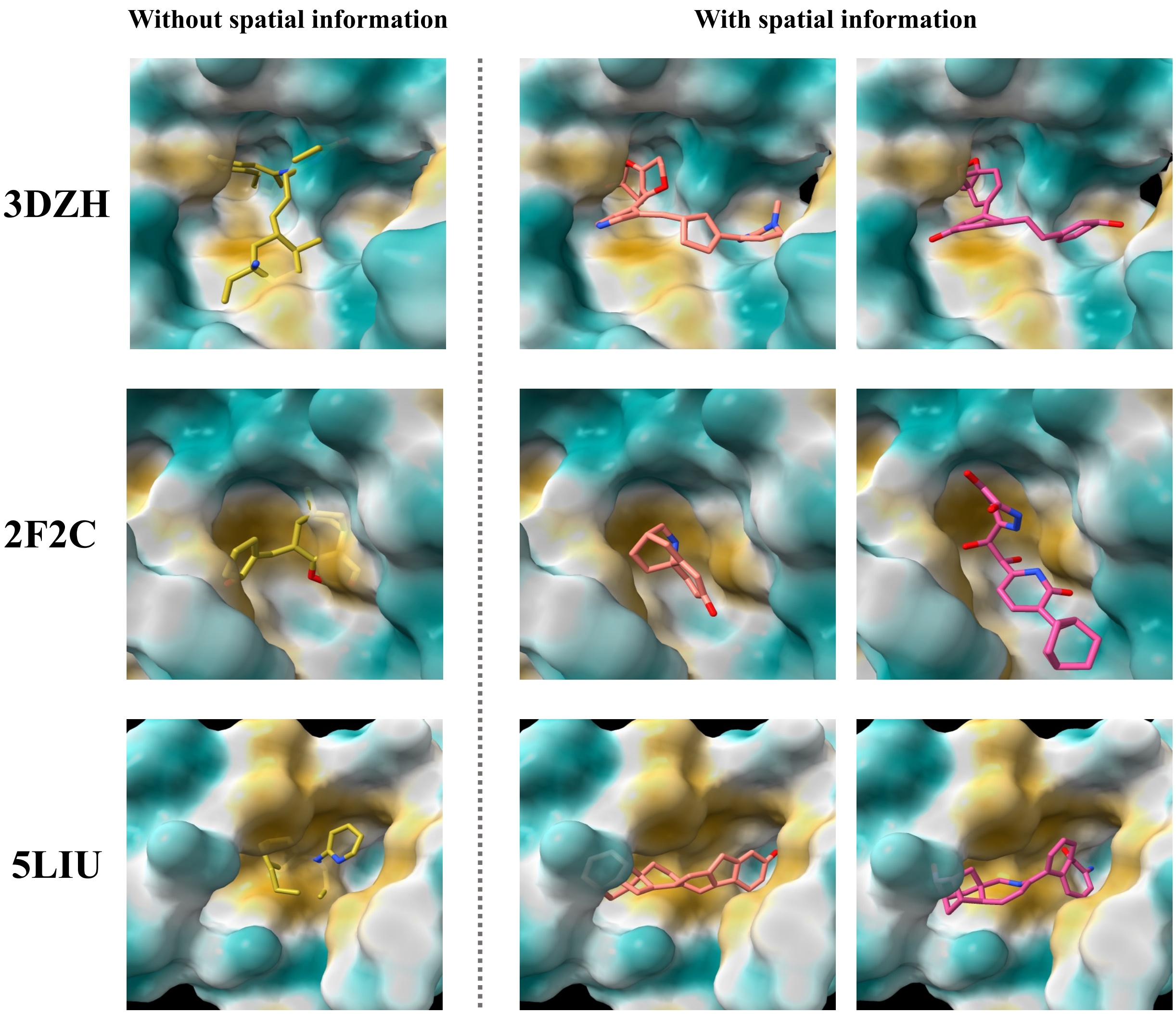

Supplementary Figure 7. Samples generated with and without conditioned spatial information.

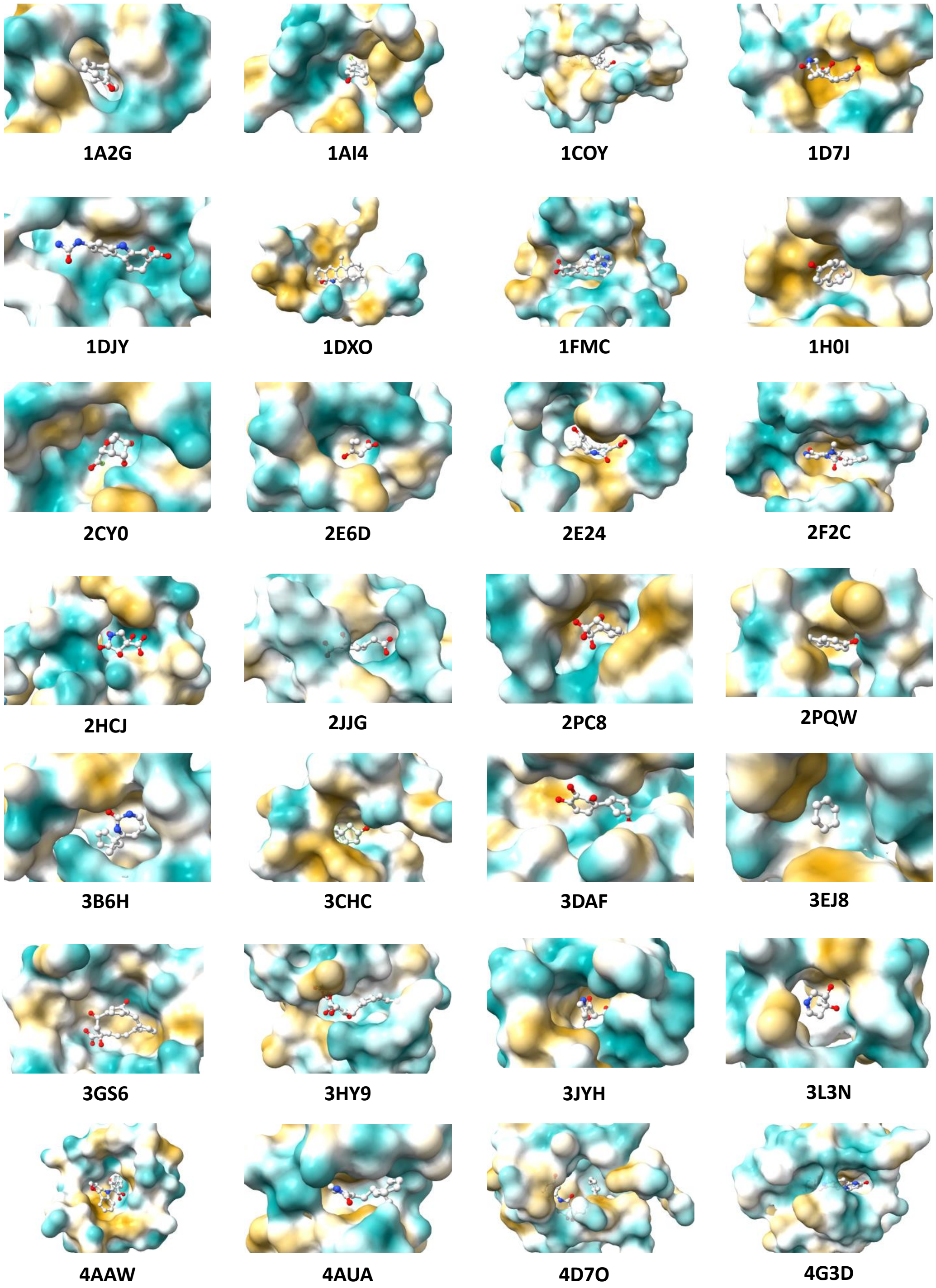

Supplementary Figure 8. Generated Samples

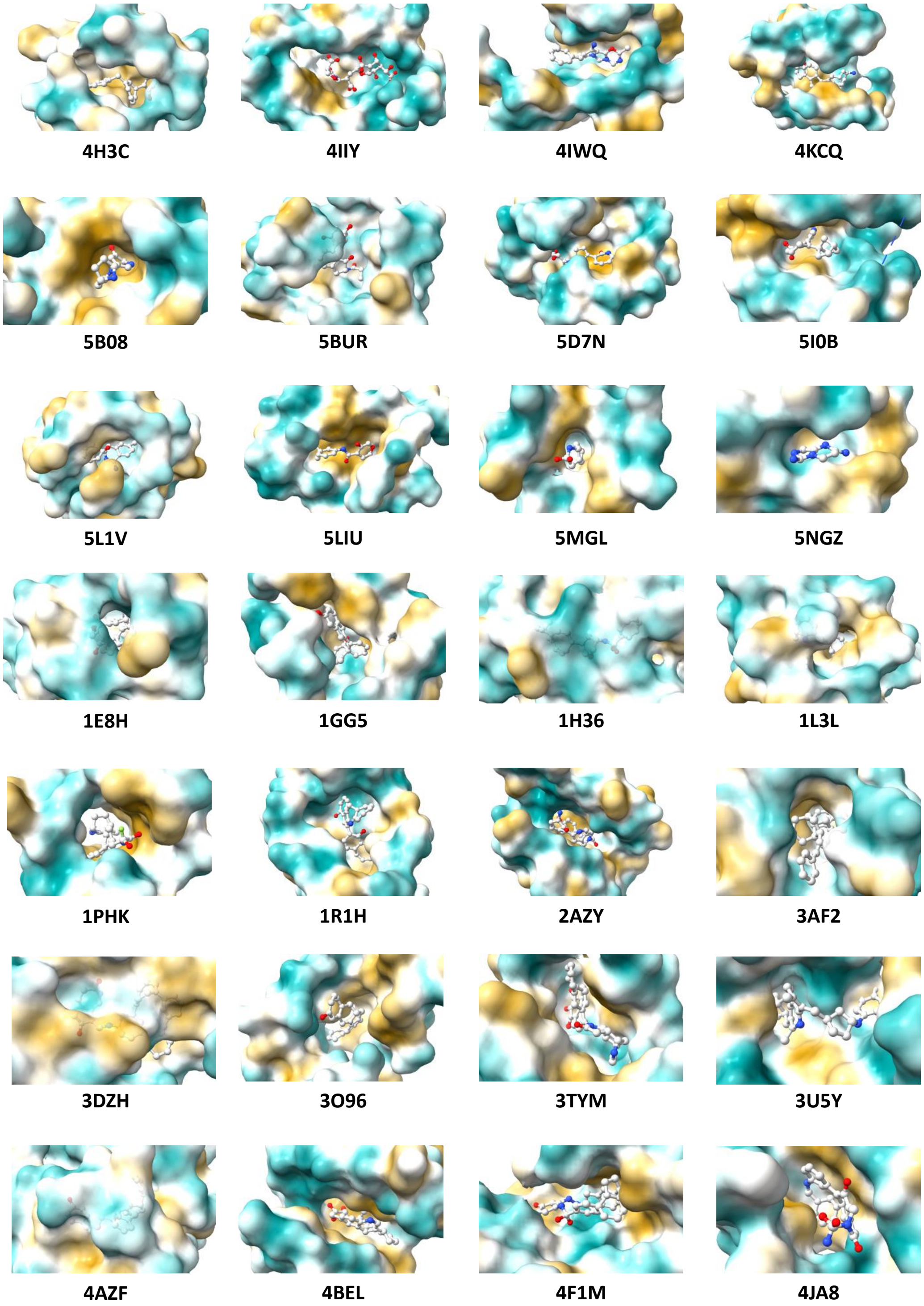

Supplementary Figure 9. Generated Samples

F Additional tables

F.1 Performance of PMDM on 1K test pairs

We retrained PMDM on 99k samples (8 V100 GPU cards for 3 days) and evaluated the performance on 1k samples which have only 20% sequence similarity. We generate 100 molecules for each protein in the test set, which takes around 4 days. The results are presented in the following table. PMDM still generates molecules with high affinities with specific proteins.

Supplementary Table 3. The results of molecules generated by PMDM on 1K test pairs.

Method	Vina Score (kcal/mol) ↓	QED ↑	SA ↑	Lipinski ↑	LogP
PMDM	-7.473±2.24	0.597±0.13	0.627±0.14	4.977±0.15	0.283±0.99
Test Set	-7.831	0.533	0.747	4.574	1.648

F.2 Chemical property results of out-of-distribution molecules

We report the chemical property values of out-of-distribution molecules in the section **Analysis of PMDM on chemical space distribution**.

Supplementary Table 4. The chemical property values of OOD molecules.

QED ↑	SA ↑	Lipinski ↑	LogP
0.448 ± 0.16	0.628 ± 0.19	4.713 ± 0.73	1.065 ± 2.22

F.3 Representative chemotypes and biochemical activities in the discovery of selective CDK2 inhibitors

We listed 12 CDK inhibitors in the following table that have been published during the discovery of CDk2 inhibitors since the 1990s. The CDK2 and CDK1 biochemical activities of the selected CDK2 inhibitors are ether tested internally using the CDK2 and CDK1 biochemical assay protocols described in this paper when they are commercially available or cited from the original publications when they are not commercially available.

Supplementary Table 5. Representative chemotypes and biochemical activities in the discovery of selective CDK2 inhibitors

Entry	Structure of CDK2 inhibitors	CDK2/E1, IC_{50} (nM)	CDK1/A2, IC_{50} (nM)	CDK1/CDK2	Entry	Structure of CDK2 inhibitors	CDK2/E1, IC_{50} (nM)	CDK1/A2, IC_{50} (nM)	CDK1/CDK2
1	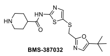 BMS-387032	21	274	13	7	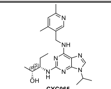 CYC065	6.4	489	76
2	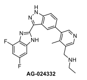 AG-024332	0.9	2.1	2.3	8[3]	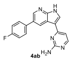 4ab	4.0	8.0	2.0
3[2]	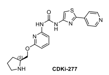 CDKi-277	4.0	8.0	2.0	9	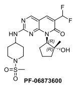 PF-06873600	0.34	4.5	13
4	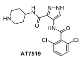 AT7519	92	172	1.9	10	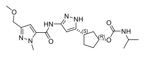 PF-07104091	3.1	117	38
5	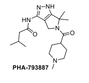 PHA-793887	5.2	55	11	11	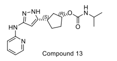 Compound 13	8.1	550	67
6	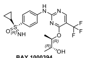 BAY 1000394	0.2	0.5	2.5	12	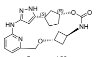 Compound 23	0.37	22	59

G Wet-lab experiments

G.1 Abbreviations

Abbreviation	Full name
DCM	1,2-Dichloromethane
DMAP	4-Dimethylaminopyridine
EtOAc	Ethyl acetate
HPLC	High-performance liquid chromatography
Prep-HPLC	Preparative High-performance liquid chromatography
LC-MS	Liquid chromatography-mass spectrometry
MeCN	Acetonitrile
NMM	N-Methylmorpholine
PE	Petroleum ether
Pd2(dba)3	Tris(dibenzylideneacetone)dipalladium(0)
Xantphos	4,5-Bis(diphenylphosphino)-9, 9-dimethylxanthene

Supplementary Table 6. Abbreviation table.

G.1.1 Experimental procedures and spectroscopic data of the selected compounds

General Procedures. All reactions were carried out under a nitrogen atmosphere with dry solvents under anhydrous conditions, unless otherwise noted. No unexpected or unusually high safety hazards were encountered. Low-resolution mass spectra (LC-MS) was used to monitor progression of reactions and was recorded on Waters ACQUITY UPLC with SQ Detectors using a Waters CORTECS C18 column (2.7 µm, 4.6 × 30 mm) using a gradient elution method: solvent A: 0.1% formic acid in water; solvent B: 0.1% formic acid in CH3CN; 5% solvent B to 95% solvent B in 1.0 min, hold 1.0 min, equilibration to 5% solvent B in 0.5 min; flow rate: 1.8 mL/min; column temperature 40 °C. Purification of final products by Prep-HPLC were carried out on Waters Prep-HPLC with QDA detector, using Xbridge C18 column (5 µm, 150 × 19 mm) using a gradient elution method. 1H NMR spectra were recorded on a Bruker Ascend 400 spectrometer. Chemical shifts are expressed in parts per million (ppm, δ units). Coupling constants are in units of hertz (Hz). Splitting patterns describe apparent multiplicities and are designated as s (singlet), d (doublet), t (triplet), q (quartet), quint (quintet), m (multiplet), br (broad). Benzyl (1-(tert-butyl)-3-((1S,3R)-3-hydroxycyclopentyl)-1H-pyrazol-5-yl)carbamate, Benzyl (1-(tert-butyl)-3-((1R,3S)-3-hydroxycyclopentyl)-1H-pyrazol-5-yl)carbamate and (trans, rac)-3-(5-(((benzyloxy)carbonyl)amino)-1-(tert-butyl)-1H-pyrazol-3-yl)cyclopentyl methanesulfonate were purchased from PharmaBlock.

G.1.2 Experiment steps for compound 9024

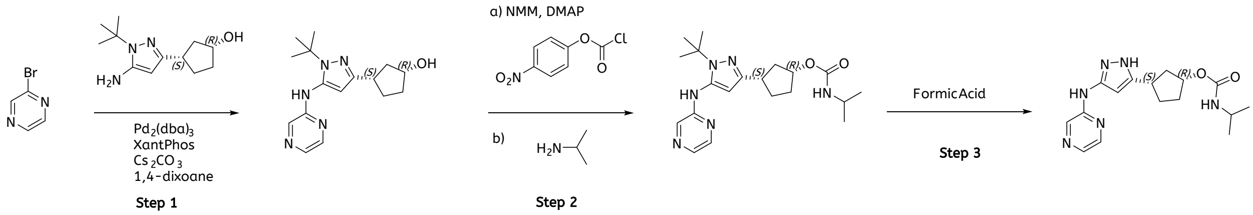

Supplementary Figure 10. Preparation of Compound 9024: (1R,3S)-3-(3-(pyrazin-2-ylamino)-1H-pyrazol-5-yl)cyclopentyl isopropylcarbamate.

Step 1: (1R,3S)-3-(1-(tert-butyl)-5-(pyrazin-2-ylamino)-1H-pyrazol-3-yl)cyclopentan-1-ol

To a solution of (1R,3S)-3-(4-amino-1-tert-butyl-pyrazol-3-yl)cyclopentanol (94.0 mg, 421 µmol) in 1,4-doioxane (5.0 mL) were sequentially added 2-bromopyrazine (100 mg, 631 µmol), Cs2CO3 (411 mg, 1.26 mmol), Pd2(dba)3 (24.4 mg, 42.1 µmol), and XantPhos (24.4 mg, 42.1 µmol) at 25 ℃. The reaction was warmed to 90 ℃ and at that temperature for 4 h. The mixture was diluted with water (20 mL) and extracted with EtOAc (30 mL× 3). The combined organic layers were washed with brine (20 mL), dried over Na2SO4, filtered and concentrated. The residue was purified by silica gel chromatography eluting with EtOAc/PE (with EtOAc from 0 to 50% in 20 min) to afford (1R,3S)-3-(1-(tert-butyl)-5-(pyrazin-2-ylamino)-1H-pyrazol-3-yl)cyclopentan-1-ol (60.0 mg, 47% yield) as a light-yellow solid. LC-MS: m/z [M+H] + calculated for C16H24N5O+ 301.2, found 302.1.

Step 2: (1R,3S)-3-(1-(tert-butyl)-5-(pyrazin-2-ylamino)-1H-pyrazol-3-yl)cyclopentyl isopropylcarbamate

To a solution of (1R,3S)-3-(1-(tert-butyl)-5-(pyrazin-2-ylamino)-1H-pyrazol-3-yl)cyclopentan-1-ol (60.0 mg, 199 µmol) in MeCN (20.0 mL) were sequentially added NMM (101 mg, 995 µmol), (4-nitrophenyl) carbonochloridate (120 mg, 597 µmol) and DMAP (48.6 mg, 398 µmol) at 25 ℃. The reaction mixture was stirred at that temperature for 3 h before isopropylamine (235 mg, 3.98 mmol) was added. The mixture was stirred at 25 ℃ for 8 h before it was concentrated under vacuum. The residue was purified by silica gel chromatography eluting with EtOAc/PE (with EtOAc from 0 to 50% in 20 min) to afford (1R,3S)-3-(1-(tert-butyl)-5-(pyrazin-2-ylamino)-1H-pyrazol-3-yl)cyclopentyl isopropylcarbamate (30.0 mg, 39% yield) as a yellow solid. LC-MS: m/z [M+H]+ calculated for C20H31N6O2+ 386.2, found 387.2.

Step 3: (1R,3S)-3-(3-(pyrazin-2-ylamino)-1H-pyrazol-5-yl)cyclopentyl isopropylcarbamate

A solution of (1R,3S)-3-(1-(tert-butyl)-5-(pyrazin-2-ylamino)-1H-pyrazol-3-yl)cyclopentyl isopropylcarbamate (30.0 mg, 77.6 µmol) in formic acid (10.0 mL) was warmed to 50 ℃ and stirred at that temperature

for 12 h. The mixture was cooled to 25 ℃ and concentrated under vacuum. The residue was purified by silica gel chromatography eluting with EtOAc/PE (with EtOAc from 0 to 80% in 25 min) to afford a crude product which was further purified by Prep-HPLC (with CH3CN from 10% to 40% in 8 min) to give (1R,3S)-3-(3-(pyrazin-2-ylamino)-1H-pyrazol-5-yl)cyclopentyl isopropylcarbamate (13.0 mg, 51% yield) as a white solid. LC-MS: m/z [M+H]+ calculated for C16H23N6O2+ 330.2, found 331.2. 1H NMR (400 MHz, DMSO-d6): δ = 12.03 (s, 1H), 9.63 (s, 1H), 8.51 (s, 1H), 8.08 (s, 1H), 7.87 (d, J = 2.8 Hz, 1H), 6.97 (d, J = 7.6 Hz, 1H), 6.21 (s, 1H), 5.00 (s, 1H), 3.60 – 3.55 (m, 1H), 3.07 – 3.03 (m, 1H), 2.48 – 2.44 (m, 1H), 2.08 – 1.99 (m, 1H), 1.92 – 1.84 (m, 1H), 1.74 – 1.57 (m, 3H), 1.03 (d, J = 6.4 Hz, 6H).

G.1.3 Experiment steps for compound 6793

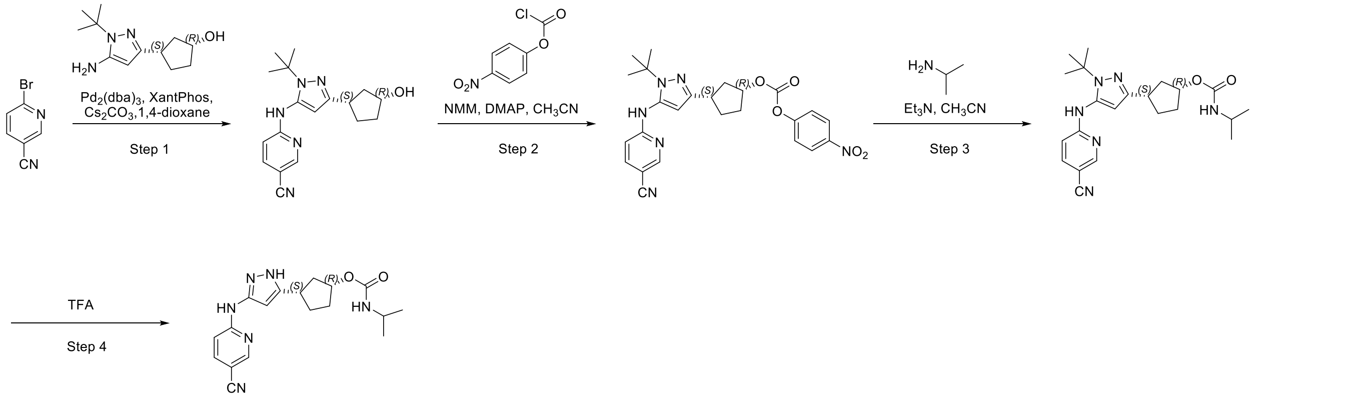

Supplementary Figure 11. Preparation of compound 6793: (1R,3S)-3-(3-((5-cyanopyridin-2-yl)amino)-1H-pyrazol-5-yl)cyclopentyl isopropylcarbamate.

Step 1: 6-((1-(tert-butyl)-3-((1S,3R)-3-hydroxycyclopentyl)-1H-pyrazol-5-yl)amino)nicotinonitrile

To a stirred solution of 6-bromopyridine-3-carbonitrile (300 mg, 1.64 mmol) in 1,4-dioxane (15.0 mL) were sequentially added (1R,3S)-3-(5-amino-1-(tert-butyl)-1H-pyrazol-3-yl)cyclopentan-1-ol (439 mg, 1.97 mmol), Pd2(dba)3 (150 mg, 164 µmol), XantPhos (95.0 mg, 164 µmol) and Cs2CO3 (1.07 g, 3.28 mmol) at 20 °C. The reaction mixture was warmed to 70 °C and stirred at that temperature for 2 h before it was cooled to 20 °C and concentrated under reduced pressure. The residue was purified by silica gel chromatography eluting with EtOAc/DCM (with EtOAc from 0 to 40% in 30 min) to give 6-((1-(tert-butyl)-3-((1S,3R)-3-hydroxycyclopentyl)-1H-pyrazol-5-yl)amino)nicotinonitrile (60.0 mg, 11% yield) as a white solid. LC-MS: m/z [M+H]+ calculated for C18H24N5O+ 326.2, found 326.2.

Step 2: (1R,3S)-3-(1-(tert-butyl)-5-((5-cyanopyridin-2-yl)amino)-1H-pyrazol-3-yl)cyclopentyl (4-nitrophenyl) carbonate

To a stirred solution of 6-((1-(tert-butyl)-3-((1S,3R)-3-hydroxycyclopentyl)-1H-pyrazol-5-yl)amino)nicotinonitrile (60.0 mg, 184 µmol) in CH3CN (5.0 mL) were sequentially added 4-methylmorpholine (101 µL, 93.3 mg, 922 µmol), (4-nitrophenyl) carbonochloridate (74.3 mg, 369 µmol) and DMAP (22.5 mg, 185 µmol) at 20 °C. The reaction mixture was stirred at that temperature for 1h before it was concentrated under reduced pressure to afford (1R,3S)-3-(1-(tert-butyl)-5-((5-cyanopyridin-2-yl)amino)-1H-pyrazol-3-yl)cyclopentyl (4-nitrophenyl) carbonate (90.0 mg, 100% yield) as a blue oil which was directly used in the next step without further purification. LC-MS: m/z [M+H]+ calculated for C25H27N6O5 + 491.2, found 491.2.

Step 3: (1R,3S)-3-(1-(tert-butyl)-5-((5-cyanopyridin-2-yl)amino)-1H-pyrazol-3-yl)cyclopentyl isopropylcarbamate

To a stirred solution of (1R,3S)-3-(1-(tert-butyl)-5-((5-cyanopyridin-2-yl)amino)-1H-pyrazol-3-yl)cyclopentyl (4-nitrophenyl) carbonate (90.0 mg, 183 µmol) in CH3CN (5.0 mL) was added propan-2-amine (78.5 µL, 54.2 mg, 917 µmol) and Et3N (128 µL, 92.8 mg, 917 µmol) at 20 °C. The resulting mixture was stirred at that temperature for 16 h before it was concentrated under reduced pressure. The residue was purified by silica gel chromatography eluting with EtOAc/PE (with EtOAc from 0% to 50% in 30 min) to give (1R,3S)-3-(1-(tert-butyl)-5-((5-cyanopyridin-2-yl)amino)-1H-pyrazol-3-yl)cyclopentyl isopropylcarbamate (65.0 mg, 86% yield) as a colorless oil. LC-MS: m/z [M+H]+ calculated for C22H31N6O2+ 411.2, found 411.2.

Step 4: (1R,3S)-3-(3-((5-cyanopyridin-2-yl)amino)-1H-pyrazol-5-yl)cyclopentyl isopropyl-carbamate

A stirred solution of (1R,3S)-3-(1-(tert-butyl)-5-((5-cyanopyridin-2-yl)amino)-1H-pyrazol-3-yl)cyclopentyl isopropylcarbamate (65.0 mg, 158 µmol) in TFA (10.0 mL) was warmed to 70 °C and stirred at that temperature for 2 h before it was cooled to 25 °C and concentrated under reduced pressure. The residue was purified by Prep-HPLC eluting with CH3CN in water (with CH3CN from 10% to 60% in 40 min) to give (1R,3S)-3-(3-((5-cyanopyridin-2-yl)amino)-1H-pyrazol-5-yl)cyclopentyl isopropylcarbamate (10.0 mg, 18% yield) as a white solid. LC-MS: m/z [M+H]+ calculated for C18H23N6O2 + 355.2, found 355.2. 1H NMR (400 MHz, DMSO- d6): δ = 12.10 (s, 1H), 10.01 (s, 1H), 8.53 (d, J = 2.0 Hz, 1H), 7.88 (dd, J = 8.8, 2.4 Hz, 1H), 7.27 (s, 1H), 6.97 (d, J = 7.6 Hz, 1H), 6.19 (s, 1H), 5.00 (s, 1H), 3.57 (dd, J = 14.0, 6.8 Hz, 1H), 3.23 – 2.95 (m, 1H), 2.49 – 2.41 (m, 1H), 2.02 (dd, J = 15.2, 7.2 Hz, 1H), 1.96 – 1.84 (m, 1H), 1.70 (t, J = 12.4 Hz, 2H), 1.60 (s, 1H), 1.10 – 0.93 (m, 6H).

G.1.4 Experiment steps for compound 6849

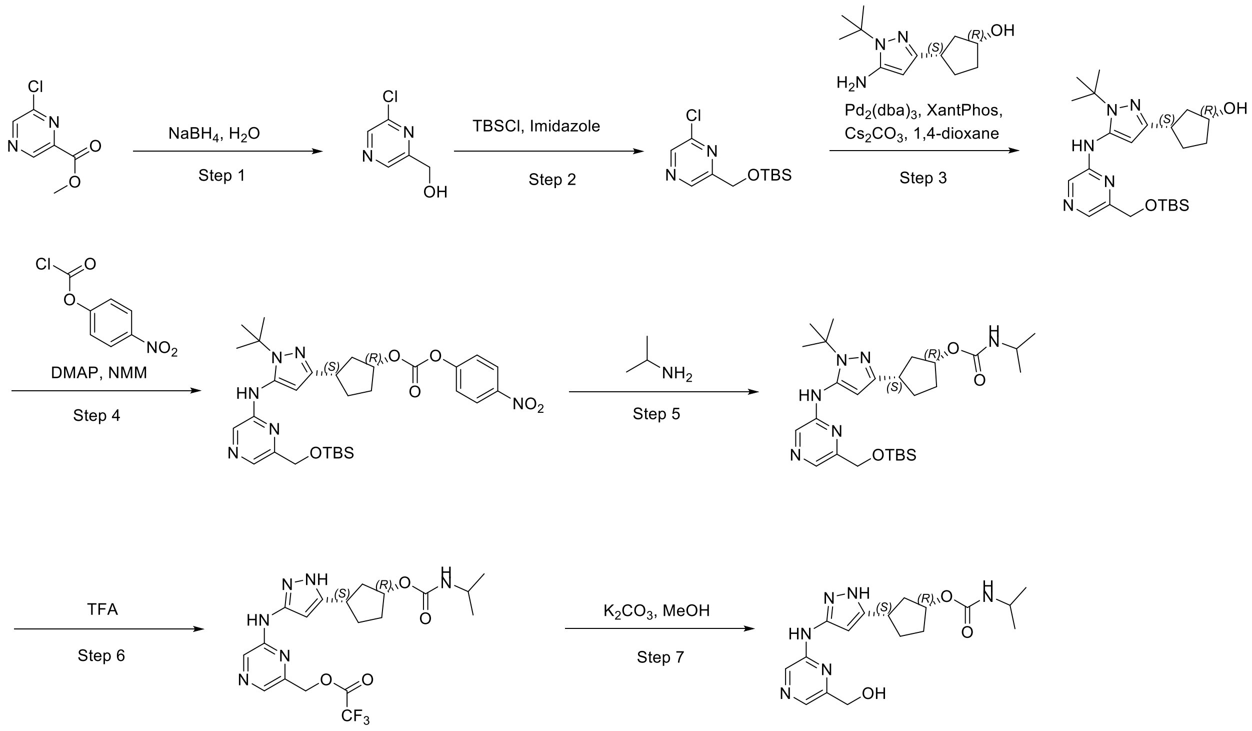

Supplementary Figure 12. Preparation of compound 6849: (1R,3S)-3-(3-((6-(hydroxymethyl)pyrazin-2-yl)amino)-1H-pyrazol-5-yl)cyclopentyl ((S)-sec-butyl)carbamate.

Step 1: (6-chloropyrazin-2-yl)methanol

To a stirred solution of methyl 6-chloropyrazine-2-carboxylate (3.50 g, 20.3 mmol) in H2O (50.0 mL) was added NaBH4 (3.85 g, 101 mmol) in portions at 0 °C. The reaction mixture was warmed to 20 °C and stirred at that temperature for 0.5 h, followed by addition of sat. aq. K2CO3 (70 mL) and EtOH (50 mL). The resulting mixture was stirred for another 1h and extracted with EtOAc (100 mL × 2). The combined organic phases were dried over Na2SO4 and then filtered. The filtrate was concentrated under reduced pressure. The residue was purified by silica gel chromatography eluting with EtOAc/petroleum ether (with EtOAc from 0 to 30% in 20 min) to give (6-chloropyrazin-2-yl)methanol (920 mg, 31% yield) as a yellow oil. LC-MS: m/z [M+H]+ calculated for C5H6ClN2O+ 145.0, found 145.2.

Step 2: 2-(((tert-butyldimethylsilyl)oxy)methyl)-6-chloropyrazine

To a stirred solution of (6-chloropyrazin-2-yl)methanol (400 mg, 2.77 mmol) and Imidazole (377 mg, 5.53 mmol) in CH2Cl2 (10.0 mL) was added TBSCl (251 mg, 3.04 mmol) at 0 °C. The reaction mixture was warmed to 20 °C and stirred at that temperature for 2 h before it was quenched with water (50 mL)

and then extracted with CH2Cl2 (50 mL × 2). The combined organic phases were dried over Na2SO4 and then filtered. The filtrate was concentrated under reduced pressure. The residue was purified by silica gel chromatography eluting with EtOAc/petroleum ether (with EtOAc from 0 to 30% in 20 min) to give 2-(((tert-butyldimethylsilyl)oxy)methyl)-6-chloropyrazine (600 mg, 84% yield) as a yellow oil. LC-MS: m/z [M+H]+ calculated for C11H20ClN2OSi+ 259.1, found 259.0.

Step 3: (1R,3S)-3-(5-amino-1-(tert-butyl)-1H-pyrazol-3-yl)cyclopentan-1-ol

To a stirred solution of 2-(((tert-butyldimethylsilyl)oxy)methyl)-6-chloropyrazine (300 mg, 1.89 mmol) in 1,4-dioxane (5.0 mL) were sequentially added (1R,3S)-3-(1-(tert-butyl)-5-((6-(((tert-butyldimethylsilyl)oxy)methyl)pyrazin-2-yl)amino)-1H-pyrazol-3-yl)cyclopentan-1-ol (338 mg, 1.51 mmol), Pd2(dba)3 (173 mg, 189 µmol), Xant-Phos (110 mg, 189 µmol) and Cs2CO3 (1.85 g, 5.68 mmol) at 25 °C. The reaction mixture was warmed to 100 °C and stirred at that temperature for 3 h. The mixture was cooled and concentrated under reduced pressure. The residue was purified by flash column chromatography eluting with petroleum ether/EtOAc (with EtOAc from 0 to 70% in 25 min) to afford (1R,3S)-3-(5-amino-1-(tert-butyl)-1H-pyrazol-3-yl)cyclopentan-1-ol (130 mg, 20% yield) as a yellow solid. LC-MS: m/z [M+H]+ calculated for C23H40N5O2Si + 446.3, found 446.1.

Step 4: (1R,3S)-3-(1-(tert-butyl)-5-((6-(((tert-butyldimethylsilyl)oxy)methyl)pyrazin-2-yl)amino)-1H-pyrazol-3-yl)cyclopentyl (4-nitrophenyl) carbonate

To a stirred solution of (1R,3S)-3-(5-amino-1-(tert-butyl)-1H-pyrazol-3-yl)cyclopentan-1-ol (100 mg, 224 µmol) in CH3CN (5.0 mL) were sequentially added 4-nitrophenyl carbonochloridate (136 mg, 673 µmol), NMM (123 µL, 113 mg, 1.12 mmol) and DMAP (54.8 mg, 449 µmol) at 20 °C. The reaction mixture was stirred at that temperature for 3 h before it was concentrated under reduced pressure to give (1R,3S)-3-(1-(tert-butyl)-5-((6-(((tert-butyldimethylsilyl)oxy)methyl)pyrazin-2-yl)amino)-1H-pyrazol-3-yl)cyclopentyl (4-nitrophenyl) carbonate (crude) as a yellow oil which was directly used in the next step without further purification. LC-MS: m/z [M+H]+ calculated for C30H43N6O6Si+ 611.3, found 611.1.

Step 5: (1R,3S)-3-(1-(tert-butyl)-5-((6-(((tert-butyldimethylsilyl)oxy)methyl)pyrazin-2-yl)amino)-1H-pyrazol-3-yl)cyclopentyl isopropylcarbamate

To a stirred solution of (1R,3S)-3-(1-(tert-butyl)-5-((6-(((tert-butyldimethylsilyl)oxy)methyl)pyrazin-2-yl)amino)-1H-pyrazol-3-yl)cyclopentyl (4-nitrophenyl) carbonate (crude) in CH3CN (5.0 mL) was added propan-2-amine (182 µL, 126 mg, 2.13 mmol) at 20 °C. The reaction mixture was stirred at that temperature for 2 h before it was concentrated under reduced pressure. The residue was purified by silica gel chromatography eluting with EtOAc/petroleum ether (with EtOAc from 0 to 100% in 20 min) to give (1R,3S)-3-(1-(tert-butyl)-5-((6-(((tert-butyldimethylsilyl)oxy)methyl)pyrazin-2-yl)amino)-1H-pyrazol-3-yl)cyclopentyl isopropy-

lcarbamate (90.0 mg, 79% yield) as a yellow oil. LC-MS: m/z [M+H]+ calculated for C27H47N6O3Si+ 531.3, found 531.2.

Step 6: (6-((5-((1S,3R)-3-((isopropylcarbamoyl)oxy)cyclopentyl)-1H-pyrazol-3-yl)amino)pyrazin-2-yl)methyl 2,2,2-trifluoroacetate

A stirred solution of (1R,3S)-3-(1-(tert-butyl)-5-((6-(((tert-butyldimethylsilyl)oxy)methyl)pyrazin-2-yl)amino)-1H-pyrazol-3-yl)cyclopentyl isopropylcarbamate (15.0 mg, 28.3 µmol) in TFA (2.0 mL) was warmed to 70 °C and stirred at that temperature for 16 h. The mixture was concentrated under reduced pressure to afford (6-((5-((1S,3R)-3-((isopropylcarbamoyl)oxy)cyclopentyl)-1H-pyrazol-3-yl)amino)pyrazin-2-yl)methyl 2,2,2-trifluoroacetate (crude) as a yellow oil. LC-MS: m/z [M+H]+ calculated for C19H24F3N6O4+ 457.2, found 457.2.

Step 7: (1R,3S)-3-(3-((6-(hydroxymethyl)pyrazin-2-yl)amino)-1H-pyrazol-5-yl)cyclopentyl isopropylcarbamate

To a stirred solution of (6-((5-((1S,3R)-3-((isopropylcarbamoyl)oxy)cyclopentyl)-1H-pyrazol-3-yl)amino)pyrazin-2-yl)methyl 2,2,2-trifluoroacetate (crude) in MeOH (2.0 mL) was added K2CO3 (3.02 mg, 21.9 µmol) at 20 °C. The reaction mixture was stirred at that temperature for 6 h before it was concentrated under reduced pressure. The residue was purified by Prep-HPLC eluting with CH3CN in water (with CH3CN from 5% to 70% in 30 min) to give (1R,3S)-3-(3-((6-(hydroxymethyl)pyrazin-2-yl)amino)-1H-pyrazol-5-yl)cyclopentyl isopropylcarbamate (3.30 mg, 42% yield) as a yellow solid. LC-MS: m/z [M+H]+ calculated for C17H25N6O3+ 361.2, found 361.1. 1H NMR (400 MHz, DMSO-d6): δ = 11.92 (s, 1H), 9.53 (s, 1H), 8.43 (s, 1H), 7.91 (s, 1H), 6.93 (d, J = 7.6 Hz, 1H), 6.11 (s, 1H), 5.38 (s, 1H), 4.96 (s, 1H), 4.44 (d, J = 4.4 Hz, 2H), 3.53 (dt, J = 13.4, 6.8 Hz, 1H), 3.06 – 2.95 (m, 1H), 2.39 (d, J = 7.6 Hz, 1H), 2.02 – 1.81 (m, 2H), 1.75 – 1.50 (m, 3H), 0.99 (d, J = 6.4 Hz, 6H).

G.1.5 Experiment steps for compound 7246

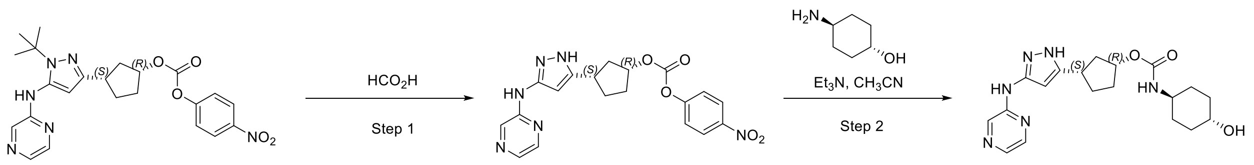

Supplementary Figure 13. Preparation of compound 7246: (1R,3S)-3-(3-(pyrazin-2-ylamino)-1H-pyrazol-5-yl)cyclopentyl ((1r,4R)-4-hydroxycyclohexyl)carbamate

Step 1: 4-nitrophenyl ((1R,3S)-3-(3-(pyrazin-2-ylamino)-1H-pyrazol-5-yl)cyclopentyl) carbonate

To a stirred solution of (1R,3S)-3-(1-(tert-butyl)-5-(pyrazin-2-ylamino)-1H-pyrazol-3-yl)cyclopentyl (4-nitrophenyl) carbonate (100 mg, 215 µmol) in HCO2H (10.0 mL) was warmed to 80 °C and stirred at that temperature for 2 h. The mixture was concentrated under reduced pressure. The crude product was used in the next step without further purification.

Step 2: (1R,3S)-3-(3-(pyrazin-2-ylamino)-1H-pyrazol-5-yl)cyclopentyl ((1r,4R)-4-hydroxycyclohexyl)carba[illegible]

To a stirred solution of 4-nitrophenyl((1R,3S)-3-(3-(pyrazin-2-ylamino)-1H-pyrazol-5-yl)cyclopentyl) carbonate in CH3CN (10.0 mL) was added 4-aminocyclohexan-1-ol (49.5 mg, 430 µmol) and Et3N (297 µL, 217 mg, 2.15 mol) at 25°C. The mixture was stirred at that temperature for 2 h. The reaction mixture was concentrated under reduced pressure. The residue was purified by Prep-HPLC eluting with CH3CN in water (with CH3CN from 5% to 40% in 30 min) to afford (1R,3S)-3-(3-(pyrazin-2-ylamino)-1H-pyrazol-5-yl)cyclopentyl ((1r,4R)-4-hydroxycyclohexyl)carbamate (22.7 mg, 27.3% yield) as a white solid. LC-MS: m/z [M+H]+ calculated for C19H27N6O3+ 387.2, found 387.3. 1H NMR (400 MHz, DMSO-d6): δ = 11.98 (s, 1H), 9.61 (s, 1H), 8.51 (s, 1H), 8.11 – 8.06 (m, 1H), 7.90 – 7.82 (m, 1H), 7.00 – 6.90 (m, 1H), 6.20 (s, 1H), 5.03 – 4.92 (m, 1H), 4.55 – 4.45 (m, 1H), 3.32 – 3.25 (m, 1H), 3.22 – 3.12 (m, 1H), 3.10 – 3.00 (m, 1H), 2.47 – 2.38 (m, 1H), 2.80 – 1.96 (m, 1H), 1.94 – 1.82 (m, 1H), 1.81 – 1.65 (m, 6H), 1.63 – 1.55 (m, 1H), 1.24 – 1.07 (m, 4H).

G.1.6 Experiment steps for compound 6216

Step 1: 3,6-dibromo-2-(bromomethyl)pyridine

A mixture of 3,6-dibromo-2-methylpyridine (10.0 g, 39.9 mmol), AIBN (6.54 g, 39.9 mmol) and NBS (14.2 g, 79.7 mmol) in CCl4 (100.0 mL) was stirred at 90 °C for 16 h before it was concentrated under reduced pressure. The residue was purified by silica gel chromatography eluting with EtOAc/PE (with EtOAc from 0 to 10% in 20 min) to give 3,6-dibromo-2-(bromomethyl)pyridine (9.00 g, 69% yield). LC-MS: m/z [M+H]+ calculated for C6H5Br3N+ 329.8, found 329.4.

Step 2: tert-butyl (3-((3,6-dibromopyridin-2-yl)methoxy)propyl)carbamate

To a stirred solution of NaH (0.67 g, 16.7 mmol, 60% purity) in THF (30.0 mL) was added tert-butyl (3-hydroxypropyl)carbamate (2.93 g, 16.7 mmol) at 0 °C. The reaction mixture was stirred at that temperature for 30 min before a solution of 3,6-dibromo-2-(bromomethyl)pyridine (5.00 g, 15.2 mmol) in THF (50.0 mL) was added. The resulting mixture was stirred at 0 °C for 1 h before it was diluted with H2O (50 mL) and warmed to 25 °C. The mixture was extracted with EtOAc (150 mL × 2). The combined organic phases were washed with brine (100 mL), dried over anhydrous Na2SO4, filtered and concentrated under reduced

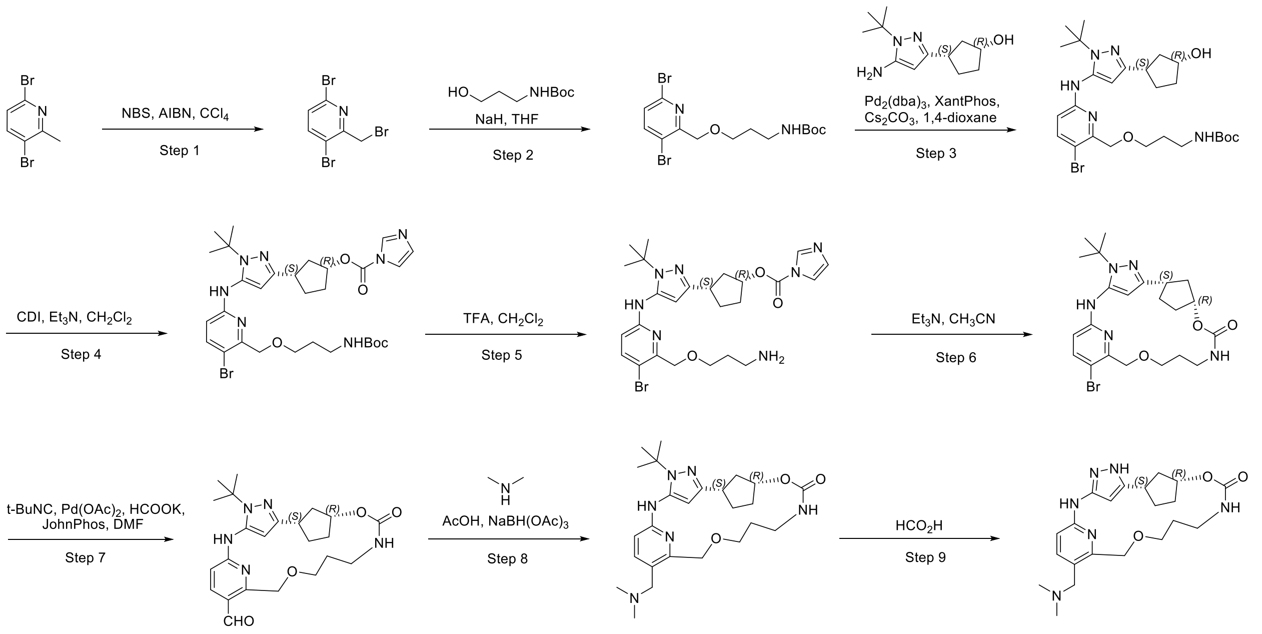

Supplementary Figure 14. Preparation of compound 6216: (11S,13R,Z)-45-((dimethylamino)methyl)-21H-6,12-dioxa-3,10-diaza-4(2,6)-pyridina-2(5,3)-pyrazola-1(1,3)-cyclopentanacyclododecaphan-11-one

pressure. The residue was purified by silica gel chromatography eluting with EtOAc/PE (with EtOAc from 0 to 50% in 20 min) to afford tert-butyl (3-((3,6-dibromopyridin-2-yl)methoxy)propyl)carbamate (2.10 g, 33% yield) as a light-yellow oil. LC-MS: m/z [M+H]+ calculated for C14H21Br2N2O3+ 425.0, found 424.9.

Step 3: tert-butyl (3-((3-bromo-6-((1-(tert-butyl)-3-((1S,3R)-3-hydroxycyclopentyl)-1H-pyrazol-5-yl)amino)pyridin-2-yl)methoxy)propyl)carbamate

To a mixture of tert-butyl (3-((3,6-dibromopyridin-2-yl)methoxy)propyl)carbamate (360 mg, 849 µmol), (1R,3S)-3-(5-amino-1-(tert-butyl)-1H-pyrazol-3-yl)cyclopentan-1-ol (190 mg, 849 µmol) and Cs2CO3 (553 mg, 1.70 mmol) in 1,4-dioxane (8.0 mL) were sequentially added XantPhos (49.2 mg, 84.9 µmol) and Pd2(dba)3 (77.8 mg, 84.9 µmol) at 25 °C. The reaction mixture was stirred at 80 °C under N2 atmosphere for 3 h before it was concentrated under reduced pressure. The residue was purified by silica gel chromatography eluting with EtOAc/PE (with EtOAc from 0 to 80% in 20 min) to afford tert-butyl (3-((3-bromo-6-((1-(tert-butyl)-3-((1S,3R)-3-hydroxycyclopentyl)-1H-pyrazol-5-yl)amino)pyridin-2-yl)methoxy)propyl)carbamate (280 mg, 58% yield) as a light-yellow oil. LC-MS: m/z [M+H]+ calculated for C26H41BrN5O4+ 566.2, found 566.6.

Step 4: (1R,3S)-3-(5-((5-bromo-6-((3-((tert-butoxycarbonyl)amino)propoxy)methyl)pyridin-2-yl)amino)-1-(tert-butyl)-1H-pyrazol-3-yl)cyclopentyl 1H-imidazole-1-carboxylate

To a stirred solution of tert-butyl (3-((3-bromo-6-((1-(tert-butyl)-3-((1S,3R)-3-hydroxycyclopentyl)-1H-

pyrazol-5-yl)amino)pyridin-2-yl)methoxy)propyl)carbamate (280 mg, 494 µmol) in CH2Cl2 (12.0 mL) were sequentially added Et3N (207 µL, 150 mg, 1.48 mmol) and CDI (213 mg, 1.48 mmol) at 25 °C. The reaction mixture was warmed to 35 °C and stirred at that temperature for 2 h before it was cooled, diluted with H2O (30 mL) and extracted with CH2Cl2 (50 mL × 2). The combined organic phase was washed with brine (20 mL) and dried over anhydrous Na2SO4, filtered and concentrated under reduced pressure. The residue was purified by silica gel chromatography eluting with EtOAc/PE (with EtOAc from 0 to 80% in 20 min) to afford (1R,3S)-3-(5-((5-bromo-6-((3-((tert-butoxycarbonyl)amino)propoxy)methyl)pyridin-2-yl)amino)-1-(tert-butyl)-1H-pyrazol-3-yl)cyclopentyl 1H-imidazole-1-carboxylate (280 mg, 85% yield) as a light-yellow solid. LC-MS: m/z [M+H]+ calculated for C30H43BrN7O5+ 660.2, found 659.6.

Step 5: (1R,3S)-3-(5-((6-((3-aminopropoxy)methyl)-5-bromopyridin-2-yl)amino)-1-(tert-butyl)-1H-pyrazol-3-yl)cyclopentyl 1H-imidazole-1-carboxylate

To a stirred solution of (1R,3S)-3-(5-((5-bromo-6-((3-((tert-butoxycarbonyl)amino)propoxy)methyl)pyridin-2-yl)amino)-1-(tert-butyl)-1H-pyrazol-3-yl)cyclopentyl 1H-imidazole-1-carboxylate (280 mg, 424 µmol) in CH2Cl2 (20.0 mL) was added TFA (97.96 µL, 145 mg, 1.27 mmol) at 25 °C. The reaction mixture was stirred at that temperature for 2 h before it was concentrated under reduced pressure to afford (1R,3S)-3-(5-((6-((3-aminopropoxy)methyl)-5-bromopyridin-2-yl)amino)-1-(tert-butyl)-1H-pyrazol-3-yl)cyclopentyl 1H-imidazole-1-carboxylate (crude) as a light-yellow oil. LC-MS: m/z [M+H]+ calculated for C25H35BrN7O3+ 660.2, found 559.5.

Step 6: (11S,13R,Z)-45-bromo-21-(tert-butyl)-21H-6,12-dioxa-3,10-diaza-4(2,6)-pyridina-2(3,5)-pyrazola-1(1,3)-cyclopentanacyclododecaphan-11-one

To a stirred solution (1R,3S)-3-(5-((6-((3-aminopropoxy)methyl)-5-bromopyridin-2-yl)amino)-1-(tert-butyl)-1H-pyrazol-3-yl)cyclopentyl 1H-imidazole-1-carboxylate (crude) in CH3CN (10.0 mL) was added Et3N (1 mL) at 25 °C . The reaction mixture was warmed to 70 °C and stirred at that temperature for 16 h. The reaction mixture was concentrated under reduced pressure. The residue was purified by silica gel chromatography eluting with EtOAc/PE (with EtOAc from 0 to 80% in 20 min) to afford (11S,13R,Z)-45-bromo-21-(tert-butyl)-21H-6,12-dioxa-3,10-diaza-4(2,6)-pyridina-2(3,5)-pyrazola-1(1,3)-cyclopentanacyclododecaphan-11-one (150 mg, 62% yield for 2 steps) as a light-yellow oil. LC-MS: m/z [M+H]+ calculated for C22H31BrN5O3+ 492.2, found 492.0.

Step 7: (11S,13R,Z)-21-(tert-butyl)-11-oxo-21H-6,12-dioxa-3,10-diaza-4(2,6)-pyridina-2(3,5)-pyrazola-1(1,3)-cyclopentanacyclododecaphane-45-carbaldehyde

To a stirred solution of (11S,13R,Z)-45-bromo-21-(tert-butyl)-21H-6,12-dioxa-3,10-diaza-4(2,6)-pyridina-

2(3,5)-pyrazola-1(1,3)-cyclopentanacyclododecaphan-11-one (150 mg, 305 µmol) and t-BuNCO (151 mg, 1.52 mmol) in DMF (1.5 mL) were sequentially added JohnPhos (9.09 mg, 30.5 µmol), Pd(OAc)2 (6.82 mg, 30.5 µmol) and HCOOK (78.7 mg, 914 µmol) at 25 ℃. The reaction mixture was warmed to 55 ℃ and stirred at that temperature for 16 h before it was cooled to 25 ℃. The mixture was poured into H2O (5 mL) and extracted with EtOAc (5 mL × 3). The combined organic layers were washed with brine (5 mL), dried over Na2SO4, filtered and concentrated. The residue was purified by Prep-TLC(EtOAc/PE = 1: 1) to give (11S,13R,Z)-21-(tert-butyl)-11-oxo-21H-6,12-dioxa-3,10-diaza-4(2,6)-pyridina-2(3,5)-pyrazola-1(1,3)-cyclopentanacyclododeca phane-45-carbaldehyde (100 mg, 75% yield) as yellow oil. LC-MS: m/z [M+H]+ calculated for C23H32N5O4+ 442.2, found 442.2.

Step 8: (11S,13R,Z)-21-(tert-butyl)-45-((dimethylamino)methyl)-21H-6,12-dioxa-3,10-diaza-4(2,6)-pyridina-2(3,5)-pyrazola-1(1,3)-cyclopentanacyclododecaphan-11-one

To a stirred solution of (11S,13R,Z)-21-(tert-butyl)-11-oxo-21H-6,12-dioxa-3,10-diaza-4(2,6)-pyridina-2(3,5)-pyrazola-1(1,3)-cyclopentanacyclododeca phane-45-carbaldehyde (100 mg, 226 µmol) and N-methylmethanamine (566 µL, 2 M, 1.13 mmol) in DCE (1.5 mL) was added AcOH(one drop) at 25 ˚C. The reaction mixture was stirred at that temperature for 1 h before NaBH(OAc)3 (96.1 mg, 453 µmol) was added. The reaction mixture was stirred at 25 ˚C for 15 h before it was poured into saturated aqueous NaHCO3 (5 mL) and extracted with CH2Cl2 (5 mL × 3). The combined organic layers were washed with brine (5 mL), dried over Na2SO4, filtered and concentrated under reduced pressure. The residue was purifired by Prep-HPLC eluting with CH3CN in water (with CH3CN from 0% to 30% in 35 min) to afford (11S,13R,Z)-21-(tert-butyl)-45-((dimethylamino)methyl)-21H-6,12-dioxa-3,10-diaza-4(2,6)-pyridina-2(3,5)-pyrazola-1(1,3)-cyclopentanacyclododecaphan-11-one (15.0 mg, 14% yield) as a white solid. LC-MS: m/z [M+H]+ calculated for C25H39N6O3+ 471.3, found 471.3.

Step 9: (11S,13R,Z)-45-((dimethylamino)methyl)-21H-6,12-dioxa-3,10-diaza-4(2,6)-pyridina-2(5,3)-pyrazola-1(1,3)-cyclopentanacyclododecaphan-11-one

A stirred solution of (11S,13R,Z)-21-(tert-butyl)-45-((dimethylamino)methyl)-21H-6,12-dioxa-3,10-diaza-4(2,6)-pyridina-2(3,5)-pyrazola-1(1,3)-cyclopentanacyclododecaphan-11-one (15 mg, 31.87 µmol) in HCO2H (1.0 mL) was warmed to 70 ℃. The reaction mixture was stirred at that temperature for 2 h before it was cooled to 25 ℃. The mixture was concentrated under reduced pressure. The residue was purified by Prep-HPLC eluting with CH3CN in water (with CH3CN from 0% to 40% in 45 min) to afford (11S,13R,Z)-45-((dimethylamino)methyl)-21H-6,12-dioxa-3,10-diaza-4(2,6)-pyridina-2(5,3)-pyrazola-1(1,3)-cyclopentanacyclododecaphan-11-one (0.70 mg, 5.2% yield) as a white solid. LC-MS: m/z [M+H]+ calculated for C21H31N6O3+ 415.2,

found 415.3. 1H NMR (400 MHz, CD3OD): δ = 8.49 (s, 0.6H), 7.59 (d, J = 8.4 Hz, 1H), 6.94 – 6.73 (m, 2H), 5.16 (d, J = 37.0 Hz, 1H), 4.74 (d, J = 11.6 Hz, 1H), 4.62 (s, 3H), 4.29 – 4.03 (m, 2H), 3.70 (t, J = 6.0 Hz, 2H), 3.44 – 3.37 (m, 0.5H), 3.08 – 3.03 (m, 0.5H), 2.76 (d, J = 19.6 Hz, 6H), 2.63 – 2.36 (m, 1H), 2.25 – 1.63 (m, 7H).

G.1.7 Experiment steps for compound 7138

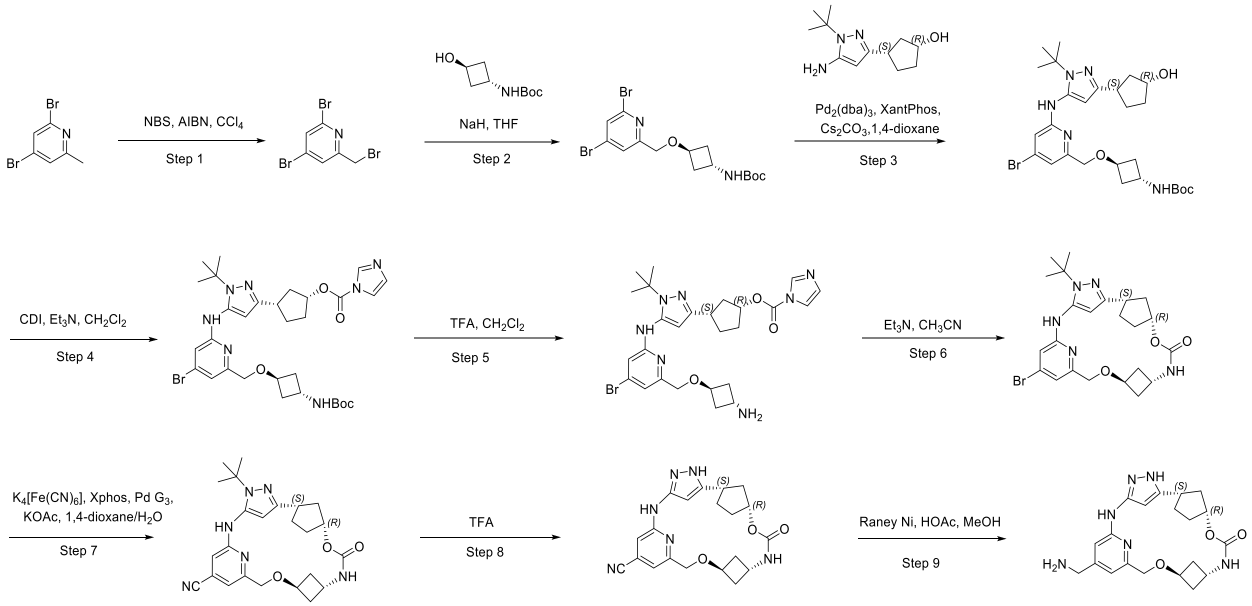

Supplementary Figure 15. Preparation of compound 7138: (11S,13R,71R,73S,Z)-9-oxo-21H-6,10-dioxa-3,8-diaza-4(2,6)-pyridina-2(5,3)-pyrazola-1(1,3)-cyclopentana-7(1,3)-cyclobutanacyclodecaphane-44-carbonitrile

Step 1: 2,4-dibromo-6-(bromomethyl)pyridine

To a stirred solution of 2-bromoisonicotinonitrile (5.00 g, 19.9 mmol) in CCl4 (50.0 mL) were sequentially added AIBN (654 mg, 3.99 mmol) and NBS (4.26 g, 23.9 mmol) at 25 °C. The reaction mixture was warmed to 80 °C and stirred at that temperature for 16 h under N2 atmosphere. The mixture was cooled and concentrated under reduced pressure. The residue was purified by silica gel chromatography eluting with EtOAc/PE (with EtOAc from 0 to 50% in 30 min) to afford 2,4-dibromo-6-(bromomethyl) pyridine (5.00 g, crude) as a yellow solid. LC-MS: m/z [M+H]+ calculated for C6H5Br3N+ 329.8, found 329.4.

Step 2: tert-butyl ((1r,3r)-3-((4,6-dibromopyridin-2-yl)methoxy)cyclobutyl)carbamate

To a stirred solution of 2,4-dibromo-6-(bromomethyl)pyridine (5.00 g, 15.2 mmol) in THF (100 mL) was added NaH (1.21 g, 60%wt. in mineral oil, 30.3 mmol) at 0 °C. The reaction mixture was stirred at 0 °C for 30 min before tert-butyl ((1r,3r)-3-hydroxycyclobutyl)carbamate (2.84 g, 15.2 mmol) was added at that temperature. The reaction mixture was warmed to 25 °C and stirred at that temperature for 16 h before it was quenched with water (200 mL) and extracted with EtOAc (200 mL × 2). The combined organic phases were dried over Na2SO4 and concentrated under reduced pressure. The residue was purified by silica gel chromatography eluting with EtOAc/PE (with EtOAc from 0 to 30% in 25 min) to afford tert-butyl ((1r,3r)-

3-((4,6-dibromopyridin-2-yl)methoxy)cyclobutyl)carbamate (2.50 g, 36% yield) as a yellow oil. LC-MS: m/z [M+Na] + calculated for $C_{15}H_{20}Br_2N_2O_3Na^+$ 460.0, found 458.8.

Step 3: tert-butyl ((1S,3r)-3-((4-bromo-6-((1-(tert-butyl)-3-((1S,3R)-3-hydroxycyclopentyl)-1H-pyrazol-5-yl)amino)pyridin-2-yl)methoxy)cyclobutyl)carbamate

To a stirred solution of tert-butyl N-[3-[(4,6-dibromo-2-pyridyl)methoxy]cyclobutyl]carbamate (2.50 g, 5.73 mmol) in 1,4-Dioxane (60.0 mL) were sequentially added (1R,3S)-3-(5-amino-1-tert-butyl-pyrazol-3-yl)cyclopentanol (1.28 g, 5.73 mmol), $Pd_2(dba)_3$ (525 mg, 0.573 mmol), XantPhos (663 mg, 1.15 mmol) and Cs_2CO_3 (3.74 g, 11.5 mmol) at 25 °C. The reaction mixture was warmed to 80 °C and stirred at that temperature for 3 h before it was cooled to 25 °C and concentrated under reduced pressure. The residue was purified by silica gel chromatography eluting with MeOH/DCM (with MeOH from 0 to 10% in 25 min) and concentrated under reduced pressure. The residue was purified by Prep-HPLC eluting with CH_3CN in water (with CH_3CN from 30% to 70% in 60 min) to give tert-butyl ((1S,3r)-3-((4-bromo-6-((1-(tert-butyl)-3-((1S,3R)-3-hydroxycyclopentyl)-1H-pyrazol-5-yl)amino)pyridin-2-yl)methoxy)cyclobutyl)carbamate(1.80 g, 52% yield) as a yellow solid. LC-MS: m/z [M+H]+ calculated for $C_{27}H_{41}BrN_5O_4^+$ 578.2, found 578.1.

Step 4: (1R,3S)-3-(5-((4-bromo-6-(((1r,3S)-3-((tert-butoxycarbonyl)amino)cyclobutoxy)methyl)pyridin-2-yl)amino)-1-(tert-butyl)-1H-pyrazol-3-yl)cyclopentyl 1H-imidazole-1-carboxylate

To a stirred suspension of tert-butyl N-[3-[[4-bromo-6-[[2-tert-butyl-5-[(1S,3R)-3-hydroxycyclopentyl]pyrazol-3-yl]amino]-2-pyridyl]methoxy]cyclobutyl]carbamate (1.71 g, 2.96 mmol) in CH_2Cl_2 (50.0 mL) were sequentially added CDI (1.28 g, 8.87 mmol), Et_3N (822 µL, 599 mg, 5.91 mmol) and DMAP (289 mg, 2.36 mmol) at 25 °C. The reaction mixture was warmed to 40 °C and stirred at that temperature for 16 h before it was cooled to 25 °C. The reaction mixture was concentrated under reduced pressure. The residue was purified by silica gel chromatography eluting with EtOAc/PE (with EtOAc from 0 to 80% in 30 min) to afford (1R,3S)-3-(5-((4-bromo-6-(((1r,3S)-3-((tert-butoxycarbonyl)amino)cyclobutoxy)methyl)pyridin-2-yl)amino)-1-(tert-butyl)-1H-pyrazol-3-yl)cyclopentyl 1H-imidazole-1-carboxylate (1.40 g, 66% yield) as a colorless oil. LC-MS: m/z [M+H]+ calculated for $C_{31}H_{43}BrN_7O_5^+$ 672.2, found 672.2.

Step 5: (1R,3S)-3-(5-((6-(((1r,3S)-3-aminocyclobutoxy)methyl)-4-bromopyridin-2-yl)amino)-1-(tert-butyl)-1H-pyrazol-3-yl)cyclopentyl 1H-imidazole-1-carboxylate

To a stirred solution of (1R,3S)-3-(5-((4-bromo-6-(((1r,3S)-3-((tert-butoxycarbonyl)amino)cyclobutoxy)methyl)pyridin-2-yl)amino)-1-(tert-butyl)-1H-pyrazol-3-yl)cyclopentyl 1H-imidazole-1-carboxylate (1.50 g, 2.23 mmol) in CH_2Cl_2 (20.0 mL) were added TFA (20.0 mL) at 25 °C. The resulting mixture was stirred at that temperature for 2 h. The mixture was concentrated under reduced pressure to give (1R,3S)-3-(5-((6-(((1r,3S)-3-

aminocyclobutoxy)methyl)-4-bromopyridin-2-yl)amino)-1-(tert-butyl)-1H-pyrazol-3-yl)cyclopentyl 1H-imidazole-1-carboxylate (1.50 g, crude) as a colorless oil. LC-MS: m/z [M+H]+ calculated for C26H35BrN7O3+ 572.2, found 572.0.

Step 6: (11S,13R,71R,73S,Z)-44-bromo-21-(tert-butyl)-21H-6,10-dioxa-3,8-diaza-4(2,6)-pyridina-2(3,5)-pyrazola-1(1,3)-cyclopentana-7(1,3)-cyclobutanacyclodecaphan-9-one

To a stirred solution of (1R,3S)-3-(5-((6-(((1r,3S)-3-aminocyclobutoxy)methyl)-4-bromopyridin-2-yl)amino)-1-(tert-butyl)-1H-pyrazol-3-yl)cyclopentyl 1H-imidazole-1-carboxylate (1.50 g, 2.62 mmol) in CH3CN (20.0 mL) was added Et3N (1.09 mL, 795 mg, 7.86 mmol) at 25 °C. The resulting mixture was stirred at that temperature for 16 h before it was concentrated under reduced pressure. The residue was purified by silica gel chromatography eluting with EtOAc/PE (with EtOAc from 0% to 80% in 30 min) to give (11S,13R,71R,73S,Z)-44-bromo-21-(tert-butyl)-21H-6,10-dioxa-3,8-diaza-4(2,6)-pyridina-2(3,5)-pyrazola-1(1,3)-cyclopentana-7(1,3)-cyclobutanacyclodecaphan-9-one (450 mg, 31% yield) as a brown solid. LC-MS: m/z [M+H]+ calculated for C23H31BrN5O3+ 504.2, found 504.0.

Step 7: (11S,13R,71R,73S,Z)-21-(tert-butyl)-9-oxo-21H-6,10-dioxa-3,8-diaza-4(2,6)-pyridina-2(3,5)-pyrazola-1(1,3)-cyclopentana-7(1,3)-cyclobutanacyclodecaphane-44-carbonitrile

To a stirred solution of (11S,13R,71R,73S,Z)-44-bromo-21-(tert-butyl)-21H-6,10-dioxa-3,8-diaza-4(2,6)-pyridina-2(3,5)-pyrazola-1(1,3)-cyclopentana-7(1,3)-cyclobutanacyclodecaphan-9-one (180 mg, 357 µmol) in 1,4-Dioxane (10.0 mL) were sequentially added Potassium hexacyanoferrate(II) trihydrate (226 mg, 535 µmol), Xphos Pd G3 (18.1 mg, 21.4 µmol), KOAc (75.5 mg, 785 µmol) and H2O (2.0 mL) at 25 °C. The reaction mixture was warmed to 100 °C and stirred at that temperature for 16 h. The mixture was cooled to 25 °C and concentrated under reduced pressure. The residue was purified by silica gel chromatography eluting with EtOAc/PE (with EtOAc from 0 to 50% in 30 min) to afford (11S,13R,71R,73S,Z)-21-(tert-butyl)-9-oxo-21H-6,10-dioxa-3,8-diaza-4(2,6)-pyridina-2(3,5)-pyrazola-1(1,3)-cyclopentana-7(1,3)-cyclobutanacyclodecaphane-44-carbonitrile (120 mg, 63% yield) as a brown solid. LC-MS: m/z [M+H]+ calculated for C24H31N6O3+ 451.2, found 451.1.

Step 8: (11S,13R,71R,73S,Z)-9-oxo-21H-6,10-dioxa-3,8-diaza-4(2,6)-pyridina-2(5,3)-pyrazola-1(1,3)-cyclopentana-7(1,3)-cyclobutanacyclodecaphane-44-carbonitrile

A solution of (11S,13R,71R,73S,Z)-21-(tert-butyl)-9-oxo-21H-6,10-dioxa-3,8-diaza-4(2,6)-pyridina-2(3,5)-pyrazola-1(1,3)-cyclopentana-7(1,3)-cyclobutanacyclodecaphane-44-carbonitrile (120 mg, 266 µmol) in TFA (10.0 mL) was warmed to 50 °C and stirred at that temperature for 3 h before it was cooled to 25 °C. The reaction mixture was concentrated under reduced pressure. The residue was purified by Prep-HPLC eluting with

CH3CN in water (with CH3CN from 30% to 70% in 40 min) to give (11S,13R,71R,73S,Z)-9-oxo-21H-6,10-dioxa-3,8-diaza-4(2,6)-pyridina-2(5,3)-pyrazola-1(1,3)-cyclopentana-7(1,3)-cyclobutanacyclodecaphane-44-carbonitrile (50.0 mg, 47% yield) as a yellow solid.LC-MS: m/z [M+H]+ calculated for C20H23N6O3+ 395.2, found 395.1.

Step 9: (11S,13R,71R,73S,Z)-44-(aminomethyl)-21H-6,10-dioxa-3,8-diaza-4(2,6)-pyridina-2(5,3)-pyrazola-1(1,3)-cyclopentana-7(1,3)-cyclobutanacyclodecaphan-9-one

To a stirred solution of (11S,13R,71R,73S,Z)-9-oxo-21H-6,10-dioxa-3,8-diaza-4(2,6)-pyridina-2(5,3)-pyrazola-1(1,3)-cyclopentana-7(1,3)-cyclobutanacyclodecaphane-44-carbonitrile (30.0 mg, 76.1 µmol) and HOAc (21.8 µL , 22.8 mg, 380 µmol) in MeOH (20.0 mL) was added Raney Ni (22.3 mg, 380 µmol) at 25°C. The reaction mixture was warmed to 50 °C and stirred at that temperature for 16 h before it was cooled to 25 °C. The reaction mixture was filtered through a pad of Celite before the filtrate was concentrated under reduced pressure. The residue was purified by Prep-HPLC eluting with CH3CN in water (with CH3CN from 30% to 70% in 40 min) to give (11S,13R,71R,73S,Z)-44-(aminomethyl)-21H-6,10-dioxa-3,8-diaza-4(2,6)-pyridina-2(5,3)-pyrazola-1(1,3)-cyclopentana-7(1,3)-cyclobutanacyclodecaphan-9-one (7.90 mg, 26% yield) as a white solid.LC-MS: m/z [M+H]+ calculated for C20H27N6O3+ 399.2, found 399.0. 1H NMR (400 MHz, DMSO-d6): δ = 9.29 (s, 1H), 7.15 (d, J = 6.8 Hz, 1H), 6.72 (s, 1H), 6.68 (s, 1H), 6.66 (s, 1H), 5.03 (d, J = 5.2 Hz, 1H), 4.30 (s, 1H), 4.12 (s, J = 9.2 Hz, 2H), 4.06 – 4.00 (m, 1H), 3.66 (s, 2H), 3.19 – 3.09 (m, 1H), 2.48 – 2.41 (m, 1H), 2.31 (d, J = 37.6 Hz, 2H), 2.12 – 1.95 (m, 3H), 1.85 (d, J = 12.0 Hz, 1H), 1.80 – 1.71 (m, 1H), 1.64 (d, J = 6.4 Hz, 2H).

G.2 Protocols of in intro assays

CDK2/Cyclin E1 assay. The LANCE Ultra time-resolved fluorescence energy transfer (TR-FRET) assay was performed to detect CDK2/Cyclin E1 catalyzed phosphorylation of peptide substrate in assay buffer containing 50 mM HEPES, pH=7.5, 10 mM MgCl2, 1mM EGTA, 2 mM DTT, 0.01% Tween, 0.1% BSA. The enzymatic reaction was carried out in a 10 µL volume containing 0.15 nM CDK2/Cyclin E1 enzyme (Carna, 04-165), 80 µM ATP, 50 nM LANCE Ultra ULight™-eIF4E-binding protein 1 (Thr37/46) Peptide (PerkinElmer, TRF0128-M) and 1% DMSO (or the test Compound at appropriate dilutions in DMSO) in the assay buffer. All the components were added to the 384-well plate (PerkinElmer, 6008280), and incubated at room temperature for 4 h. The reaction was terminated by addition of 10 µL detection buffer (PerkinElmer, CR97-100) containing 20 mM EDTA and 4 nM LANCE® Ultra Europium-anti-phospho-eIF4E-bindingprotein 1 (Thr37/46) (PerkinElmer, TRF0216-M) antibody. After 1 h of incubation at room temperature, plate was loaded on Envision Reader (PerkinElmer, EnVision Multilabel Reader) to measure

fluorescence intensity which was used to determine IC50 values of the test articles.

CDK1/Cyclin A2 assay. The LANCE Ultra time-resolved fluorescence energy transfer (TR-FRET) assay was performed to detect CDK1/Cyclin A2 catalyzed phosphorylation of peptide substrate in assay buffer containing 50 mM HEPES, pH=7.5, 10 mM MgCl2, 1mM EGTA, 2 mM DTT, 0.01% Tween, 0.1% BSA. The enzymatic reaction was carried out in a 10 µL volume containing 0.4 nM CDK1/Cyclin A2 enzyme (SignalChem, C22-18G), 10 µM ATP, 20 nM LANCE Ultra ULight™-eIF4E-binding protein 1 (Thr37/46) Peptide (PerkinElmer, TRF0128-M)) and 1% DMSO (or the test Compound at appropriate dilutions in DMSO) in the assay buffer. All the components were added to the 384-well plate (PerkinElmer, 6008280), and incubated at room temperature for 2 h. The reaction was terminated by addition of 10 µL detection buffer (PerkinElmer, CR97-100) containing 20 mM EDTA and 4 nM LANCE® Ultra Europium-anti-phospho-eIF4E-bindingprotein 1 (Thr37/46) (PerkinElmer, TRF0216-M) antibody. After 1h of incubation at room temperature, plate was loaded on Envision Reader (PerkinElmer, EnVision Multilabel Reader) to measure fluorescence intensity which was used to determine IC50 values of the test articles.

CDK9/Cyclin T1 assay. The LANCE Ultra time-resolved fluorescence energy transfer (TR-FRET) assay was performed to monitor CDK9/Cyclin T1-catalyzed phosphorylation of peptide substrate. The enzymatic reaction was carried out in a 10 µL volume containing 10 nM CDK9/Cyclin T1 enzyme (Carna, 04-110), 10 µM ATP, 50 nM Ultra ULight™-MBP substrate (PerkinElmer, TRF0109) in the assay buffer (50 mM HEPES, PH=7.5, 10 mM MgCl2, 1mM EGTA, 2 mM DTT, 0.01% Tween). All the components were added to the 384-well plate and incubated at room temperature for 2 h. The reaction was terminated by addition of 10 µL detection buffer containing 20 mM EDTA and 4 nM Ultra Europium-anti-phospho-MBP antibody (PerkinElmer, TRF0201). After 1 h of incubation at room temperature, the plate was loaded on Envision Reader to measure fluorescence intensity which was used to determine IC50 values of the test articles.

GSK3β assay. The LANCE Ultra time-resolved fluorescence energy transfer (TR-FRET) assay was performed to monitor GSK3β-catalyzed phosphorylation of peptide substrate. The enzymatic reaction was carried out in a 10 µL volume containing 1 nM GSK3β enzyme (Carna, 04-141), 10 µM ATP, 5 nM Ultra ULight™-eIF4E-binding protein 1 (Thr37/46) peptide (PerkinElmer, TRF0128) and 1% DMSO (or the test articles at appropriate dilutions in DMSO) in the assay buffer (50 mM HEPES, PH=7.5, 10 mM MgCl2, 1mM EGTA, 2 mM DTT, 0.1 mg/mL BSA, 0.01% Tween). All the components were added to the 384-well plate and incubated at room temperature for 1h. The reaction was terminated by addition of 10 µL detection buffer containing 20 mM EDTA and 4 nM Ultra Europium-anti-phospho-eIF4E-binding protein 1

(Thr37/46) antibody (PerkinElmer, TRF0216). After 1h of incubation at room temperature, the plate was loaded on Envision Reader to measure fluorescence intensity which was used to determine IC50 values of the test articles.

G.3 Representative dose-response curves of the reported compounds

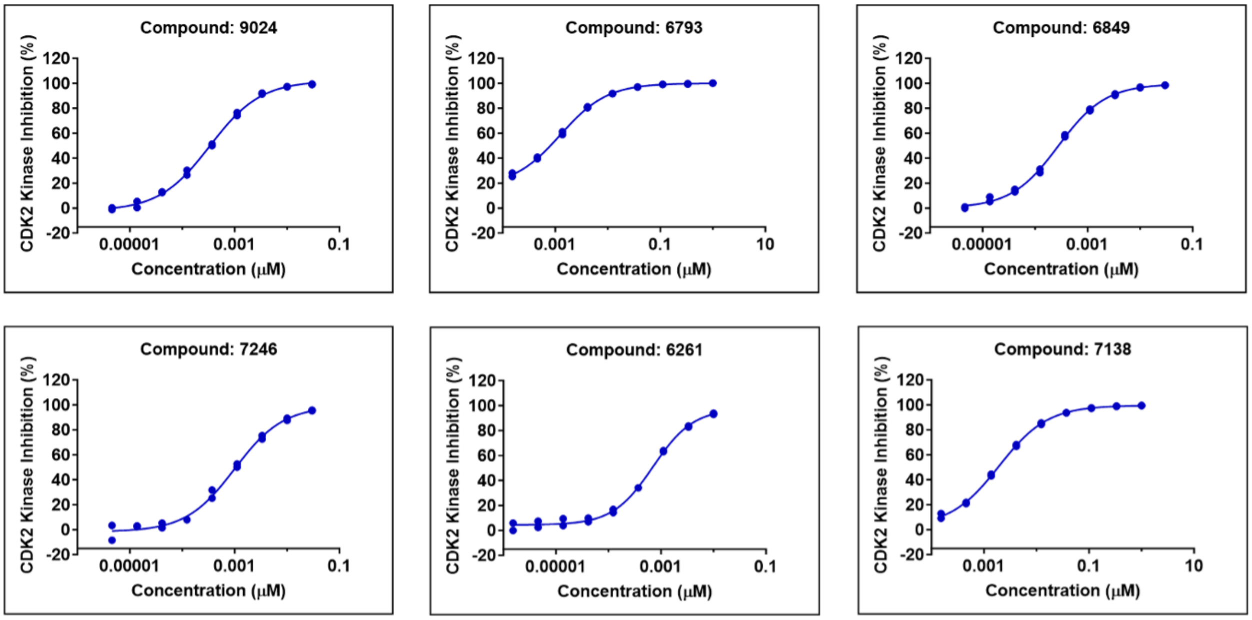

Supplementary Figure 16. Representative dose-response curves of the reported compounds to inhibit the kinase activity of CDK2/E1. Source data are provided with this paper.

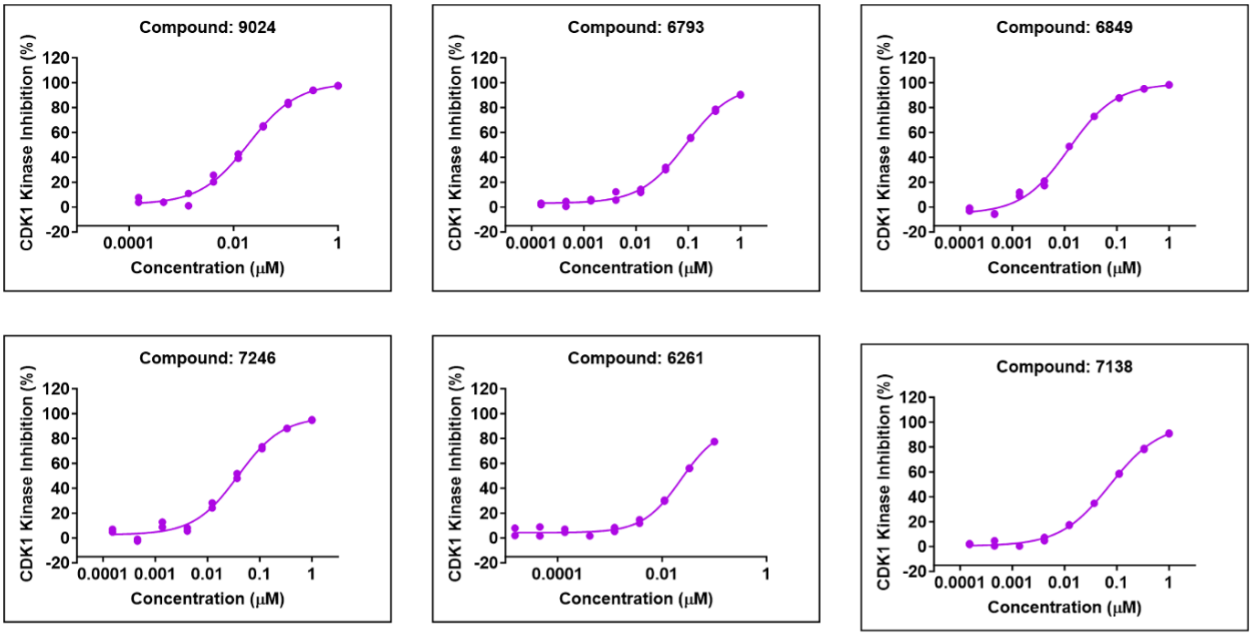

Supplementary Figure 17. Representative dose-response curves of the reported compounds to inhibit the kinase activity of CDK1/A2. Source data are provided with this paper.

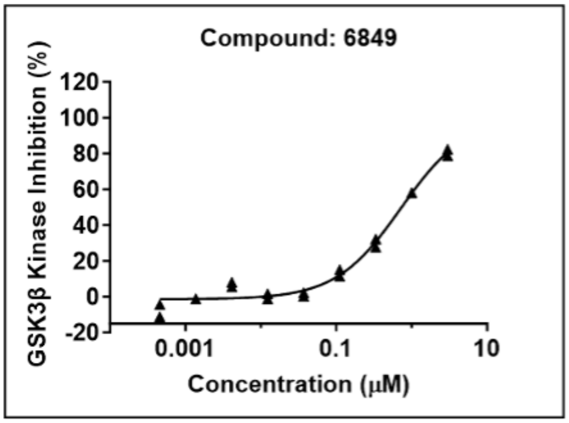

Supplementary Figure 18. Representative dose-response curves of Compound 6849 to inhibit the kinase activity of GSK3β. Source data are provided with this paper.

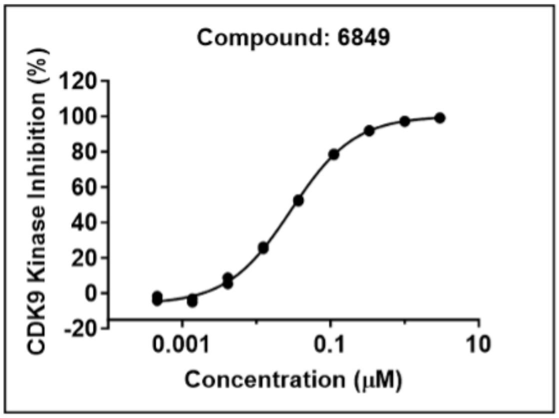

Supplementary Figure 19. Representative dose-response curves of Compound 6849 to inhibit the kinase activity of CDK9/T1. Source data are provided with this paper.

Supplementary References

[1] Jonathan Ho, Ajay Jain, and Pieter Abbeel. Denoising diffusion probabilistic models. *Advances in Neural Information Processing Systems*, 33:6840–6851, 2020.

[2] Jonas Köhler, Leon Klein, and Frank Noé. Equivariant flows: exact likelihood generative learning for symmetric densities. In *International conference on machine learning*, pages 5361–5370. PMLR, 2020.

[3] Chence Shi, Shitong Luo, Minkai Xu, and Jian Tang. Learning gradient fields for molecular conformation generation. In *International Conference on Machine Learning*, pages 9558–9568. PMLR, 2021.
