# Supplementary material for: A dual diffusion model enables 3D molecule generation and lead optimization based on target pockets
Source: Nat Commun. 2024 Mar 26;15:2657. doi: 10.1038/s41467-024-46569-1 (PMC10965937; doi:10.1038/s41467-024-46569-1)
Supplement: Supplementary file 3 — Reporting Summary [file 41467_2024_46569_MOESM3_ESM.pdf]

nature portfolio

Corresponding author(s): Hailong Li, Ka-Chun Wong, Hengtong Zhang

Last updated by author(s): Feb 7, 2024

Reporting Summary

Nature Portfolio wishes to improve the reproducibility of the work that we publish. This form provides structure for consistency and transparency in reporting. For further information on Nature Portfolio policies, see our Editorial Policies and the Editorial Policy Checklist.

Statistics

For all statistical analyses, confirm that the following items are present in the figure legend, table legend, main text, or Methods section.

n/a	Confirmed	
☐	☒	The exact sample size (*n*) for each experimental group/condition, given as a discrete number and unit of measurement
☒	☐	A statement on whether measurements were taken from distinct samples or whether the same sample was measured repeatedly
☒	☐	The statistical test(s) used AND whether they are one- or two-sided<br>*Only common tests should be described solely by name; describe more complex techniques in the Methods section.*
☒	☐	A description of all covariates tested
☒	☐	A description of any assumptions or corrections, such as tests of normality and adjustment for multiple comparisons
☐	☒	A full description of the statistical parameters including central tendency (e.g. means) or other basic estimates (e.g. regression coefficient) AND variation (e.g. standard deviation) or associated estimates of uncertainty (e.g. confidence intervals)
☒	☐	For null hypothesis testing, the test statistic (e.g. *F*, *t*, *r*) with confidence intervals, effect sizes, degrees of freedom and *P* value noted<br>*Give P values as exact values whenever suitable.*
☒	☐	For Bayesian analysis, information on the choice of priors and Markov chain Monte Carlo settings
☒	☐	For hierarchical and complex designs, identification of the appropriate level for tests and full reporting of outcomes
☒	☐	Estimates of effect sizes (e.g. Cohen's *d*, Pearson's *r*), indicating how they were calculated

Our web collection on statistics for biologists contains articles on many of the points above.

Software and code

Policy information about availability of computer code

Data collection	No software was used for data collection.
Data analysis	Python (version 3.9.15) and the deep learning platform PyTroch (version 1.9) were used to build the model and analyze the data.<br>QuickVina (2.1.0) was used to compute the Vina score.<br>Python Package RdKit (2021.09.5) was used to compute the chemical properties of molecules.<br>Openbabel (3.1.1) was used to build and modify the molecule file.<br>ChimeraX (1.5) was used to visualize the targeted pocket and ligand and conduct hydrogen bond analysis.<br>We used Align-it (1.0.4) for pharmacophore model analysis.<br>The source code, documentation, and instruction of PMDM are available at GitHub repository: https://github.com/Layne-Huang/PMDM/tree/main and PMDM is also released at Zenodo: https://zenodo.org/records/10631358.

For manuscripts utilizing custom algorithms or software that are central to the research but not yet described in published literature, software must be made available to editors and reviewers. We strongly encourage code deposition in a community repository (e.g. GitHub). See the Nature Portfolio guidelines for submitting code & software for further information.

Data

Policy information about availability of data

All manuscripts must include a data availability statement. This statement should provide the following information, where applicable:

- Accession codes, unique identifiers, or web links for publicly available datasets
- A description of any restrictions on data availability
- For clinical datasets or third party data, please ensure that the statement adheres to our policy

The data we used in this study has been deposited in the CrossDocked dataset: https://bits.csb.pitt.edu/files/crossdock2020/. The PDB file of SARS-CoV-2 main protease and Cyclin-dependent Kinase 2 (CDK2) has been deposited in the PDB dataset: https://www.rcsb.org under accession code 7L11 and 8H6T. The data generated in this study and processed training and test data have been publicly deposited to Zenodo under https://zenodo.org/records/10630921.

Research involving human participants, their data, or biological material

Policy information about studies with human participants or human data. See also policy information about sex, gender (identity/presentation), and sexual orientation and race, ethnicity and racism.

Reporting on sex and gender	NA
Reporting on race, ethnicity, or other socially relevant groupings	NA
Population characteristics	NA
Recruitment	NA
Ethics oversight	NA

Note that full information on the approval of the study protocol must also be provided in the manuscript.

Field-specific reporting

Please select the one below that is the best fit for your research. If you are not sure, read the appropriate sections before making your selection.

☒ Life sciences ☐ Behavioural & social sciences ☐ Ecological, evolutionary & environmental sciences

For a reference copy of the document with all sections, see nature.com/documents/nr-reporting-summary-flat.pdf

Life sciences study design

All studies must disclose on these points even when the disclosure is negative.

Sample size	Sample data were obtained from public repositories. Sample size was determined by previous published work which was used for our dataset. We choose to generate 40,000 molecules for SARS-CoV-2 main protease since 40,000 is large enough to filter molecules while we only generate 100 molecules for the targets in the test set. Similarly, we generate 10,000 and 5000 molecules for CDK2 since we only generate the scaffolds and linkers which the remaining fragments are fixed.
Data exclusions	No data were excluded from the analysis.
Replication	For each target, 100 samples were generated. Codes for the training and evaluation of the proposed method have been provided for reproducibility. Attempts at replication were successful for all results reported in the study.
Randomization	Not applicable - no experimental groups were assigned.
Blinding	No blinding was included in this study because computational data analysis was automated and unbiased.

Reporting for specific materials, systems and methods

We require information from authors about some types of materials, experimental systems and methods used in many studies. Here, indicate whether each material, system or method listed is relevant to your study. If you are not sure if a list item applies to your research, read the appropriate section before selecting a response.

Materials & experimental systems

n/a	Involved in the study
☒	☐ Antibodies
☒	☐ Eukaryotic cell lines
☒	☐ Palaeontology and archaeology
☒	☐ Animals and other organisms
☒	☐ Clinical data
☒	☐ Dual use research of concern
☒	☐ Plants

Methods

n/a	Involved in the study
☒	☐ ChIP-seq
☒	☐ Flow cytometry
☒	☐ MRI-based neuroimaging

Plants

Seed stocks	*Report on the source of all seed stocks or other plant material used. If applicable, state the seed stock centre and catalogue number. If plant specimens were collected from the field, describe the collection location, date and sampling procedures.*
Novel plant genotypes	*Describe the methods by which all novel plant genotypes were produced. This includes those generated by transgenic approaches, gene editing, chemical/radiation-based mutagenesis and hybridization. For transgenic lines, describe the transformation method, the number of independent lines analyzed and the generation upon which experiments were performed. For gene-edited lines, describe the editor used, the endogenous sequence targeted for editing, the targeting guide RNA sequence (if applicable) and how the editor was applied.*
Authentication	*Describe any authentication procedures for each seed stock used or novel genotype generated. Describe any experiments used to assess the effect of a mutation and, where applicable, how potential secondary effects (e.g. second site T-DNA insertions, mosiacism, off-target gene editing) were examined.*
